# Genetic alterations of *SUGP1* mimic mutant-*SF3B1* splice pattern in lung adenocarcinoma and other cancers

**DOI:** 10.1038/s41388-020-01507-5

**Published:** 2020-10-14

**Authors:** Samar Alsafadi, Stephane Dayot, Malcy Tarin, Alexandre Houy, Dorine Bellanger, Michele Cornella, Michel Wassef, Joshua J. Waterfall, Erik Lehnert, Sergio Roman-Roman, Marc-Henri Stern, Tatiana Popova

**Affiliations:** 1 Institut Curie, Translational Research Department, PSL Research University, Paris, France; 2 Institut Curie, INSERM U830, DNA Repair and Uveal Melanoma (D.R.U.M.), Equipe labellisée par la Ligue Nationale Contre le Cancer, PSL Research University, Paris, France; 3 Institut Curie, PSL Research University, Sorbonne University, Paris, France; 4 U934 INSERM, UMR3215 CNRS, Paris, France; 5 Seven Bridges Genomics, Konigesberg, MA USA

**Keywords:** Cancer genetics, RNA splicing

## Abstract

Genes involved in 3′-splice site recognition during mRNA splicing constitute an emerging class of oncogenes. *SF3B1* is the most frequently mutated splicing factor in cancer, and SF3B1 mutants corrupt branchpoint recognition leading to usage of cryptic 3′-splice sites and subsequent aberrant junctions. For a comprehensive determination of alterations leading to this splicing pattern, we performed a pan-TCGA screening for SF3B1-specific aberrant acceptor usage. While the most of aberrant 3′-splice patterns were explained by *SF3B1* mutations, we also detected nine *SF3B1* wild-type tumors (including five lung adenocarcinomas). Genomic profile analysis of these tumors identified somatic mutations combined with loss-of-heterozygosity in the splicing factor *SUGP1* in five of these cases. Modeling of *SUGP1* loss and mutations in cell lines showed that both alterations induced mutant-*SF3B1*-like aberrant splicing. Our study provides definitive evidence that genetic alterations of *SUGP1* genocopy *SF3B1* mutations in lung adenocarcinoma and other cancers.

## Introduction

Large-scale genomics studies have identified recurrent somatic mutations in genes encoding components of the pre-messenger RNA splicing machinery (spliceosome) in a variety of human malignancies [1]. The spliceosome is a dynamic complex containing five snRNAs (U1, U2, U4, U5, and U6) and >150 proteins that orchestrates the accurate intron recognition, excision, and exons ligation to form mature mRNA [2]. Catalog of splicing factors with frequent and recurrent somatic mutations in tumors include *SF3B1*, *U2AF1*, and *SRSF2* with heterozygous missense mutations and *ZRSR2* with loss of function mutations [3–7]. Mutations in these genes are mutually exclusive and present in up to half of myelodysplasia [3, 4], in chronic lymphocytic leukemia [7, 8] and in a significant number of solid tumors, including uveal melanoma (UM) [9–11], lung adenocarcinoma [12], and other malignancies [13, 14]. Cancer mutations of U1 spliceosomal small nuclear RNA (snRNA RNU1) were recently found in about 50% of the SHH medulloblastoma subtype and extremely rare in other types of tumors [15]. Recurrent mutations were reported in other splicing genes, including *PHF5A*, *RBM10*, and *FUBP1*, putatively implicated in cancer [16].

All cancer splicing factors are involved in the earliest stage of spliceosome assembly (spliceosome E and A complexes), where RNA and protein components work together to identify the 5′ splice site (ss), the 3′ss and the branchpoint region, with intervening polypyrimidine tract of nascent pre-mRNA [17]. Exact structural basis and order of early spliceosome assembly events remain partially understood and cancer splice mutations may contribute in identifying key genes help leading investigation of splicing machinery and indicate functional sites of proteins [2].

SF3B1, a core subunit of U2 component, is critical for branchpoint recognition and for the early stages of spliceosome assembly [18], and the most frequently mutated splicing gene in cancers [16, 19]. A key function of SF3B1 is to stabilize a duplex between the U2 snRNA and a consensus branchpoint (BP) sequence. Missense mutations found in *SF3B1* map to the surface of the HEAT-repeat domains (H3–H6) in the region that interacts with the intron between the BP and 3′ss with the hotspots at codon positions R625, K666, and K700 [20]. These mutations results in usage of cryptic BP and cryptic 3′ss typically located 10–30 nts (nucleotides) upstream of the canonical 3′ss [5, 6, 21]. Beside hotspots, other residues in H3–H6 were associated to aberrant splicing and all of them are predicted to be spatially close to one another [8, 20].

Recently, Zhang et al. demonstrated that cancer-associated mutations of *SF3B1* mainly disrupt SF3B1 interaction with SUGP1 during BP recognition and that the loss of this interaction solely accounts for the splicing errors caused by *SF3B1* mutations [22].

We started from a large-scale analysis of the TCGA series of 3′ss splicing aberrations associated with *SF3B1* mutations, questioning if all aberrant pattern has a corresponding *SF3B1* mutation. We detected tumors with an aberrant splicing pattern, which were wild-type for *SF3B1*, and found that these tumors were recurrently mutated for *SUGP1*. We further demonstrated that *SUPG1* alterations mimic the 3′ss aberration pattern found in the *SF3B1*-mutant context.

## Results and discussion

### Large-scale in silico screening for *SF3B1*^mut^ splice pattern in tumors

For a comprehensive view of pathogenic mutations inducing usage of cryptic 3′ss as observed in a *SF3B1*-mutant context (we will denote by *SF3B1*^mut^ all *SF3B1* mutations that lead to aberrant 3′ss usage), we used a Sequence Bloom Tree (SBT) [23, 24] constructed from RNA-seq data for a total of 11,350 different samples and 33 tumor types from TCGA (Supplementary Fig. S1). SBT is an indexing structure developed for querying large databases for short-read sequences. It is a fast and highly sensitive approach without false negative calls. We tested occurrence of 1443 aberrant junctions previously associated with *SF3B1*^mut^ in two independent analyses [5, 6]. The SBT score represents the number of these junctions found at least once in raw RNA-seq data (fastq) and characterizes burden of *SF3B1*^mut^ associated junctions in a tumor.

**Fig. 1:**
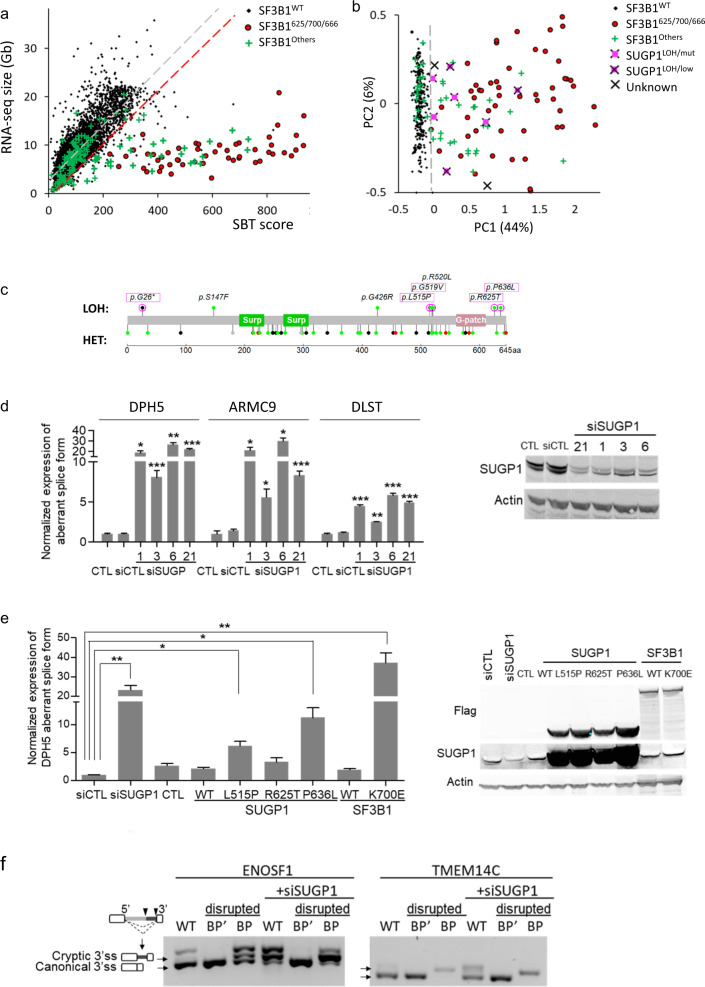
SF3B1-like pattern in the TCGA and SUGP1. **a** Screening RNA-seq data in the TCGA using the SBT score. The SBT score (the occurrence of 1443 aberrant splice junctions in fastq RNA-seq) for each sample is plotted (*x*-axis) against the size of RNA-seq bam file (*y*-axis). Cases with *SF3B1* hotspot (red points) and other mutations (green crosses) are indicated. The linear trend (gray) and the cutoff (red) lines for the cases further explored are shown. **b** Principal component analysis of the selected 456 cases (including cases with high SBT scores and control tumor cases) characterized by the splicing index (SI) in 366 cryptic 3′ss junctions selected in an unsupervised way (see “Materials and methods”). Cases with *SUGP1* alterations are highlighted (magenta spots). The two first principal components, PC1 and PC2, are shown and the fraction of variance explained is indicated. The cutoff for PC1 scores to discriminate cases with SF3B1-like phenotype was set to median + 3·MAD (Median Absolute Deviation) (gray dashed line). **c** Mutations found in *SUGP1* in all tumor types (TCGA). Missense, nonsense, frameshift, and splice mutations are indicated by green, black, red, and gray points, respectively. Mutations with loss of heterozygosity (LOH) and in heterozygous state are shown above and below the protein representation, respectively. Mutations associated with a 3′ss aberrant pattern are highlighted by magenta frames. **d** Effect of siRNA-mediated knockdown of *SUGP1*, on the aberrant splice forms of *DPH5, DLST*, and *ARMC9* in the HEK293T cell line. Relative expression of cryptic 3′ss junction normalized to the canonical 3′ss junction was determined by quantitative RT–PCR, and effect of the different siRNA #1, 3, 6, and 21 was compared with the control (CTL) (paired *t* test; *p < 0.05; **p < 0.005; ***p < 0.0005). The protein knockdown was confirmed by immunoblotting with anti-SUGP1, using β-actin as a loading control. **e** Effect of siRNA-mediated knockdown of *SUGP1*, overexpression of wild-type *SUGP1* or *SF3B1*, overexpression of *SUGP1*^L515P, R625T or P636L^ or *SF3B1*^K700E^ on the aberrant splice form of *DPH5* in HEK293T cell line. Relative expression of cryptic 3′ss junction normalized to the canonical 3′ss junction of *DPH5* was determined by quantitative RT–PCR. The results are average of three replicates and are represented as mean ± sd, and each condition is compared to the control (paired *t* test; *p < 0.05; **p < 0.005). The protein knockdown or overexpression was confirmed by immunoblotting with anti-Flag and anti-SUGP1 using β-actin as a loading control. **f** Minigene splice assay of two *SF3B1*^mut^-sensitive 3′ss (*ENOSF1, TMEM14C*) and their cryptic (BP’) and canonical (BP) branchpoint mutants. Gel electrophoresis shows the different splicing processes for minigene ExonTrap constructions in *SF3B1*^WT^ cell line HEK293T with or without siRNA-mediated knockdown of *SUGP1*. The lower band corresponds to the usage of the canonical 3′ss. The intermediate band corresponds to the usage of the cryptic 3′ss. The upper band corresponds to the heteroduplex formation from the two products.

After adjustment for RNA-seq coverage, the 138 top SBT-score cases were selected following the cutoff determined by the lowest SBT score of a validated *SF3B1*^A633V^ case (Supplementary Table 1, Fig. 1a). These high SBT-score cases were thoroughly verified for *SF3B1* mutations in exome and RNA sequencing data using IGV [25].

We detected six additional *SF3B1*-mutated cases not previously reported [16]. Six cases with marginal variant allele frequency (VAF) at a subclonal level (RNA-seq VAF~0.1) displayed high SBT scores. Cases showing no read supporting an *SF3B1* hotspot mutation in the RNA-seq data had consistently low SBT scores. *SF3B1* mutations at positions 742, 741, and 633 represented the lower limit of SBT scores observed in tumors mutated for *SF3B1* in the 500–800aa region. Two UCEC cases displayed identical mutations p.R549C and elevated SBT scores, possibly defining the N-terminal limit (domain H1) of functional sites for which mutations could induce 3′ss aberrations.

To confirm the aberrant splicing pattern, we analyzed 3′ss usage in 456 cases, including cases identified by a high SBT score (*n* = 128) and a selection of control cases using the STAR software (“Materials and methods”, Supplementary Table 1). We calculated quantiles of canonical and aberrant junctions, denoted CQ and AQ, respectively. The set of 366 3′ss aberrant junctions was selected from ubiquitously-expressed exons (CQ ≥ 0.3 in 95% cases) in an unsupervised manner: the junction was included if aberrant and corresponding canonical junction were expressed (AQ ≥ 0.4 and CQ ≥ 0.5) and splicing index SI ≥ 0.15 at least in one sample. Principal component analysis (PCA) of cryptic 3′ss usage characterized by SI showed the main source of variation to be *SF3B1* mutations and the phenotype is characterized by increased expression of cryptic 3′ss (Fig. 1b, Supplementary Fig. S2a). We considered 83 cases, including 74 *SF3B1*-mutated cases, to display an aberrant splice pattern, on the ground that their PC1 (principal component 1) scores exceeded the median + 3·MAD (Median Absolute Deviations) cutoff (Supplementary Table 1). This result represents the exhaustive list of the TCGA cases with 3′ss aberrations characteristic to *SF3B1* hotspots mutations (Supplementary Fig. S2b).

### *SUGP1* alterations associated with a *SF3B1*^mut^ splice pattern in tumors

Nine tumors showing high levels of the 3′ss pattern but not mutated in *SF3B1* (hereafter named SF3B1-like) were detected, including lung adenocarcinomas (LUAD, five cases), hepatocellular carcinoma (LIHC, one case), mesothelioma (MESO, one case), acute myeloid leukemia (LAML, one case), and skin melanoma (SKCM, one case).

Mutational analysis of RNA processing genes (GO:0006396) of the nine SF3B1-like cases revealed mutations in *SUGP1* (also known as *Splicing Factor 4* or *SF4*) as the only common event for five cases: four missense (p.L515P, p.G519V, p.R625T, and p.P636L) and one stop-gain (p.G26*) mutations. Further analyses using SNP-arrays revealed loss of heterozygosity (LOH) of the *SUGP1* locus in all five cases and VAF in RNA-seq data was consistent with loss of the wild-type allele (Supplementary Table 1). Given that only eight cases out of the entire TCGA series carried *SUGP1* variants with RNA-seq VAF > 0.3 and LOH, enrichment of *SUGP1* variant + LOH (*SUGP1*^LOH/mut^) within cases with a SF3B1-like phenotype is highly significant (*p* < 10^–8^, Fisher’s exact test adjusted for multiple testing). Worth noting that beside five *SUGP1* mutations + LOH associated with an SF3B1-like phenotype, there are three missense mutations + LOH and more than 50 missense and deleterious mutations in heterozygous state found in the TCGA (Fig. 1c). We reviewed the splicing pattern found in the corresponding tumors and confirmed the absence of any enrichment in 3′ss aberrations (Supplementary Fig. S3a).

We further mined the four SF3B1-like cases associated with neither *SF3B1* nor *SUGP1* mutation. Normalized *SUGP1* expression levels in two cases (one LUAD and one LIHC) were the lowest in the corresponding cohorts having *z*-scores < −5 and expression at the lower limit for expressed genes (Supplementary Fig. S4bc). Interestingly, we also observed LOH in the *SUGP1* locus for these two cases. The remaining two cases (one LAML and one SKCM) associated with the SF3B1-like splice pattern were not found altered for *SUGP1*. Of note, the LAML case with a strong SF3B1-like pattern harbored a *U2AF1*^S34Y^ hotspot mutation, which is not likely to be causal, as 20 other *U2AF1*^S34Y/F^ cases showed no evidence of such pattern and as *U2AF1* mutations are known to drive an alternative exon usage [26, 27].

### SUGP1^LOH/mut^ display aberrant 3′ splicing and cryptic branchpoint recognition

The splicing factor SUGP1 has two SURP and one G-patch domains and was shown to be dispensable for the assembly of a functional splicing complex [28, 29]. SUGP1 interacts with SF3B1 and *SF3B1* hotspot mutations disrupt this interaction. Moreover, aberrant recognition of BPs and cryptic 3′ss was shown for *SUGP1* KD and for double mutations in G-patch domain of SUGP1 [22]. Here we detected five mutations in *SUGP1* associated with LOH and SF3B1-like splicing pattern in cancers (Fig. 1c). Interestingly, missense mutations do not target any known interaction domain of SUGP1 and are located before and after its G-patch domain.

We assessed the impact of *SUGP1* alterations found in cancers, including LOH and mutations, on splicing. Using HEK293T cells (wild-type for both *SUGP1* and *SF3B1)*, we performed siRNA-mediated *SUGP1* knockdown and overexpression of the *SUGP1* L515P, R625T, and P636L mutants. As readout, we assessed the splicing ratio, which is the ratio of aberrant to canonical junction expression, in three *SF3B1*^mut^–sensitive junctions: *DPH5*, *DLST* and *ARMC9*, as previously reported [5]. The knockdown of *SUGP1* using four different siRNAs consistently and significantly induced the *SF3B1*^mut^-aberrant pattern (*p* < 0.05 to <0.0005, depending on the siRNA and junctions; Fig. 1d). Transiently overexpressed *SUGP1* mutants induced either significant but modest effects on splicing (L515P and P636L) or no significant effect (R625T) (Fig. 1e; Supplementary Fig. S3b), arguing against strong dominant-negative properties of these mutants.

The p.G26* stop mutation, located at the very beginning of the gene, was well expressed in the tumor, suggesting an alternative initiation of translation bypassing the Nonsense-Mediated mRNA Decay (NMD) in this sample (Supplementary Fig. S4b). Indeed, we demonstrated by expressing *SUGP1* cDNA carrying this G26* mutation the existence of this alternative initiation coding an N-terminal truncated protein (Supplementary Fig. S4c).

In an SF3B1-mutant context, the U2 complex has a preferential recognition for the cryptic branchpoint BP’. To determine whether SUGP1 loss affects U2 recognition of the BP in a similar manner, we performed a splice-reporter assay with *SF3B1*^mut^-sensitive junctions (*ENOSF1* and *TMEM14C*) containing adenine mutants inactivating either the canonical or cryptic BPs [5]. Our results showed that mutants disrupting the BP’ abolish the splice aberration induced by siRNA-mediated *SUGP1* knockdown (Fig. 1f). This finding demonstrates that SUGP1 is critical for correct recognition of the BP by the U2 complex, and that its loss leads to aberrant 3′ss usage similar to *SF3B1*^mut^.

### Common splice pattern of *SUGP1* and *SF3B1 cases* in LUAD

**Fig. 2:**
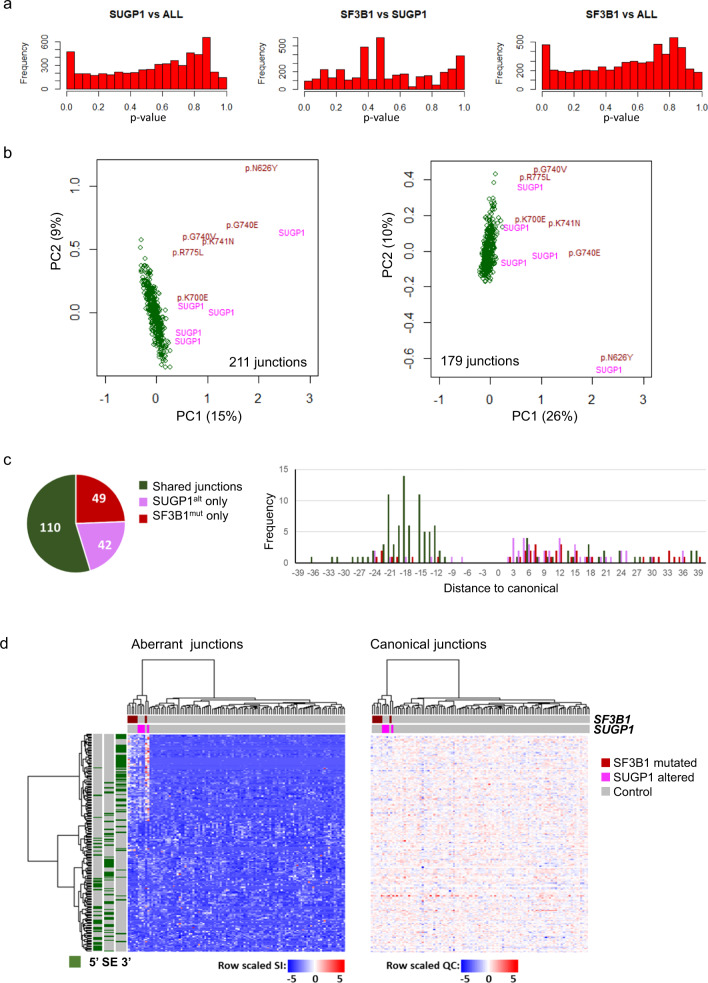
Global similarity of splice alteration patterns in *SUGP1* and *SF3B1*. **a** *P* value distribution in *SUGP1*^alt^ vs. Controls (left panel), *SF3B1*^mut^ vs. Controls (right panel), and *SUGP1*^alt^ vs. *SF3B1*^mut^ (central panel) comparisons in the LUAD series performed by Wilcoxon rank test on the splicing index (SI) of 3′ss proximal aberrant junctions. **b** Principal component 2D plot for the set of aberrant 3′ss junctions selected independently for *SUGP1*^alt^ vs. Controls (left panel) and *SF3B1*^mut^ vs. Controls (right panel) comparisons. *SUGP1*^alt^ and *SF3B1*^mut^ cases are indicated. **c** Further analysis of the set of junctions from (**b**): 201 junctions (selection from union of 211 and 179) were classified as shared, *SF3B1*^mut^-specific and *SUGP1*^alt^-specific based on the maximal SI in a group. Pie diagram (left panel, counts are indicated) and distribution of aberrant junction positions (right panel, *x* = 0 corresponds to canonical exon start) are shown. **d** Hierarchical clustering and the heatmap of differentially spliced cryptic 3′ss junctions (*n* = 127) with high loadings, which separate *SUGP1*^alt^ and *SF3B1*^mut^ from the Control group in PCA, combined with cryptic 5′ss junctions (*n* = 45) and aberrant exon usage (*n* = 47) popped up in the comparisons but not separating mutated and control cases in the LUAD cohort (left panel). Cases are annotated according to their alterations in *SF3B1* or *SUGP1*, 100 controls for visualization are selected randomly; type of aberrant junctions are indicated in the side row panels. For comparison, canonical junction expression is shown (increased expression of aberrant junctions is not explained by canonical junction expression; right panel).

We questioned overall impact of cancer *SUGP1* alterations (*SUGP1*^alt^) on splicing in comparison to *SF3B1*^mut^, taking advantage of LUAD cohort containing five *SUGP1* (four *SUGP1*^LOH/Mut^ and one case with the lowest *SUGP1* expression) and six *SF3B1*^mut^ cases; cases without mutations in splicing genes were taken as controls (400 LUAD cases). We developed a bioinformatics protocol to test similarity of the global aberrant splicing patterns, which takes into account transcriptional heterogeneity of lung adenocarcinoma, significant variation in RNA-seq coverage and small sample sizes of *SUGP1*^alt^ and *SF3B1*^mut^ groups (“Materials and methods”). First, we applied Wilcoxon nonparametric rank test to compare SI in *SUGP1*^alt^ vs. Controls, *SF3B1*^mut^ vs. Controls and *SUGP1*^alt^ vs. *SF3B1*^mut^ for each type of splice aberration. *P* value distribution in both *SUGP1*^alt^ vs. Controls and *SF3B1*^mut^ vs. Controls comparisons showed significant enrichment in low *p* values only for the proximal 3′ss (*p* value < 10^−11^, Fisher test; Fig. 2a, Supplementary Figs. S5–S8). *SUGP1*^alt^ vs. *SF3B1*^mut^ did not show any enrichment in low *p* values for any type of splice aberration considered, evidencing no differences beyond background (Fig. 2a, Supplementary Figs. S5–S8).

Second, we applied PCA for each comparison in each type of splice aberrations using corresponding junctions with Wilcoxon test *p* values < 0.05 (not corrected for multiple testing), to test if there is a major (with proportion of explained variance >5%) principal component (PC) that separates *SUGP1*^alt^, *SF3B1*^mut^, and the Control group. If such PCs are found, aberrant junctions with high loadings (correlated with PC) are picked up as differentially expressed aberrant junctions.

Comparison of proximal 3′ss cryptic junctions revealed PCs associated to *SUGP1*^alt^ and *SF3B1*^mut^. Moreover, (i) the junctions correlated with PCs (211 and 179, respectively) were largely shared (110 in common, *p* < 10^–10^); and (ii) *SUGP1*^alt^ were not found separated from *SF3B1*^mut^ in major PCs subspace: at least one case with *SUGP1*^alt^ has *SF3B1*^mut^ as a nearest neighbor and vice versa (Fig. 2b). Figure 2c shows a refined analysis of 238 (shared and definitively not shared, see “Materials and methods”) aberrant junctions and their distribution around exon start (−50 to 50nts selection). Shared junctions constitute 85% of aberrant AG’ at 10–30nts distance upstream exon start, evidencing that *SUGP1*^alt^ and *SF3B1*^mut^ have a common set of sensitive splice sites (Fig. 2c). Consequently, the nucleotide composition of aberrant acceptors in *SF3B1*^*mut*^ and *SUGP1*^*alt*^ as analyzed by Weblogo showed no difference (Supplementary Fig. S9a).

Following the same approach, we explored 5′ss proximal, 3′ and 5′ss distant aberrations and alternative exon usage. No major PC separating *SUGP1*^alt^ and *SF3B1*^mut^ from the Control group was found in any of comparisons (Supplementary Figs. S5–S8). Even though the differences between *SF3B1*^mut^ and *SUGP1*^alt^ could be noticed, they are not specific to *SF3B1*^mut^ and *SUGP1*^alt^, because the aberrations are shared by some (or many) control cases, which is further illustrated by the heatmap (Fig. 2d). Hierarchical clustering on the aberrant junctions with high loadings demonstrates background level in cryptic 5′ss and aberrant exon usage in both *SUGP1*^alt^ and *SF3B1*^mut^ groups. Conversely, alternative junctions using cryptic 3′ss displayed overexpression in both *SUGP1*^alt^ and *SF3B1*^mut^ groups as compared with wild-type tumors (Fig. 2d). Furthermore, cryptic 3′ss were mostly found 10–30nts upstream to canonical 3′ss in both groups, with a strong concurrence of sensitive junctions (Fig. 2c). Therefore, we conclude that we observed no difference in aberrant splicing pattern between *SF3B1*^mut^ and *SUGP1*^alt^ in the LUAD cohort.

Here we applied supervised PCA protocol that helps revealing relevant junctions even with moderate significance, eliminating effect of confounding factors. The condition for PC selection to separate Control and *SF3B1*^mut^ or *SUGP1*^alt^ groups accounts for transcriptional heterogeneity and follows the assumption that aberrant junctions show up almost exclusively in the groups with alteration in a splicing factor (if many controls behave similar to mutated cases, the feature is not specific to the mutated cases even though the test shows significant differences (Supplementary Figs. S5–S8).

Exploring the possible common consequences of *SF3B1*^mut^ or *SUGP1*^alt^ in oncogenesis, we found three common altered genes belonging to the cancer gene census (https://cancer.sanger.ac.uk/census) (Supplementary Fig. S9b). Among previously described targets *PPP2R5A* [30] was found in both contexts, whereas *BRD9* [31] was only targeted in a *SF3B1*^mut^ context. Only a minority (47 out of 110) of aberrant junctions led to out-of-frame transcripts when 2/3 were expected, suggesting that the majority of out-of-frame junctions used in a *SF3B1*^mut^ or *SUGP1*^alt^ context are not detected, probably because degraded by NMD process (Supplementary Fig. S9с).

### SF3B1^mut^-splice pattern in *SUGP1*^alt^ cellular models

To evaluate if *SUGP1* alterations are indeed the causes of the abnormal splicing pattern, we set up two experimental models using HEK293T and isogenic HAP1 cell lines and analyzed their RNA-seq data.

**Fig. 3:**
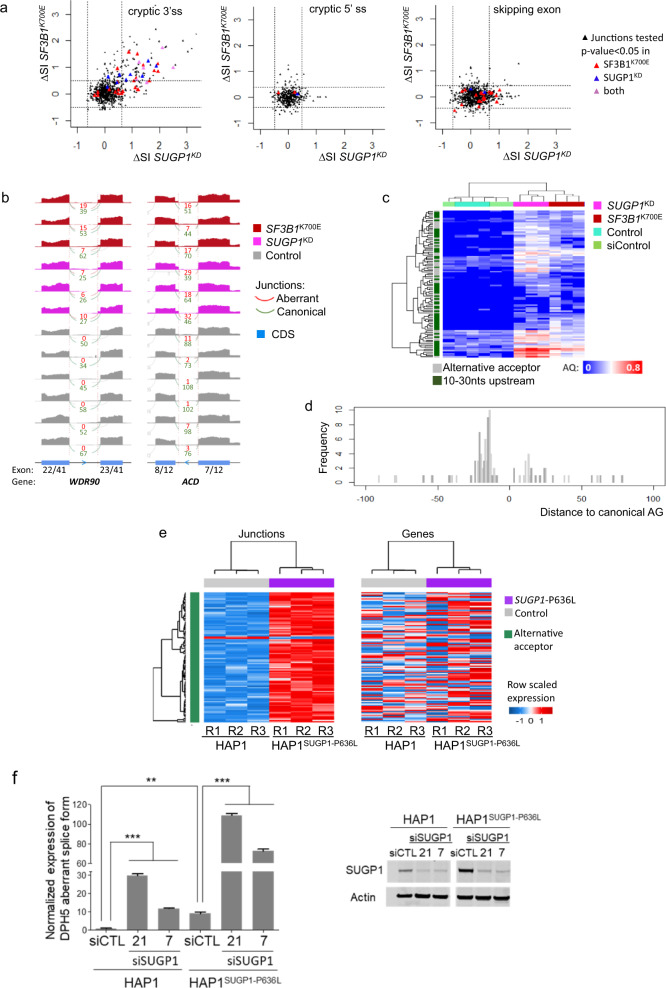
SF3B1-like splice pattern analysis in HEK293T and HAP1^SUGP1-P636L^ isogenic cell line. **a** Relative splicing index (∆SI_max_) in *SUGP1*-depleted (*SUGP1*^*KD*^) HEK293T cells (*x*-axis) and HEK293T cells over-expressing *SF3B1*^K700E^ (*y*-axis) in different types of splicing defects: 3′ss aberration (left panel), 5′ss aberration (central panel), and aberrant exon usage (right panel). Junctions were selected in semi-unsupervised way (“Materials and methods”). Dotted lines indicate 2·MAD (Median Absolute Deviation), junctions significantly different (*p* value < 0.05) in Student *t* test comparison of mean values are marked by red (*SUGP1*^*KD*^), blue (*SF3B1*^K700E^) or violet (both) dots. **b** Sashimi plots showing read counts in two junctions found aberrant in experimental models *SUGP1*-depleted (*SUGP1*^*KD*^), over-expressing *SF3B1*^K700E^, and Controls HEK293T cells: chr16:708344-708509-708524 (left panel) and chr16:67692719-67692735-67692830 (right panel). Aberrant (red) and canonical (green) split reads counts are indicated. **c** Hierarchical clustering and heatmap of aberrant 3′ss junctions with ∆SI_max_ > 1 in either *SUGP1*^KD^ or *SF3B1*^K700E^ (74 and 49 junctions, respectively, 97 in total) HEK293T cell lines. Raw quantiles of aberrant junction split-read counts are shown. **d** Distances between the cryptic and canonical 3′ss for 97 junctions with ∆SI_max_ > 1 in either *SUGP1*^KD^ or *SF3B1*^K700E^. The position of the canonical 3′ss is set to 0. **e** Hierarchical clustering and heatmap of differential splice 3′ss junctions (*p* value ≤ 0.05, Log2FC ≥ 1) in HAP1 and HAP1^SUGP1-P636L^ isogenic cell lines. Three biological replicates for each cell line (R1–R3) are indicated below the heatmap. The corresponding gene level expression heatmap is shown on the right panel for comparison (differential expression of junctions is not the consequence of differential gene expression). **f** siRNA-mediated knockdown *of SUGP1*^WT^ and *SUGP1*^P636L^ impact on *DPH5* aberrant junction expression in HAP1 isogenic cell lines. Relative expression of cryptic 3′ss junction normalized to the canonical 3′ss junction of *DPH5* was determined by quantitative RT–PCR. The results are average of three replicates and are represented as mean ± sd, and each condition is compared to the control (paired *t* test; **p < 0.005; ***p < 0.0005). The protein knockdown was confirmed by immunoblotting with anti-SUGP1 using β-actin as a loading control.

Splicing patterns in HEK293T cells transiently depleted for *SUGP1* (SUGP1^KD^) or over-expressing *SF3B1*^K700E^ were compared with siControl and *SF3B1*^WT^ overexpression, using relative shift in splice indexes (∆SI_max_) (“Materials and methods”). We selected junctions in a semi-unsupervised way and visualized data using 2D plots showing relative change in splice indexes *SUGP1*^KD^ vs. *SF3B1*^K700E^ for each junction (Fig. 3a). This showed coherent changes in *SUGP1*^KD^ and *SF3B1*^K700E^ splice profiles only within 3′ss aberrations (Pearson’s correlation *r* = 0.6, *p* value < 10^–10^; two illustrative examples are shown in Fig. 3b). Aberrations at 5′ss as well as alternative exon usage showed single outlying junctions with high ∆SI_max_ and no evidence of being systematically affected in either of experimental groups (Supplementary Figs. S10–S13). The subset of aberrant junctions with ∆SI_max_ > 1 in either *SUGP1*^KD^ or *SF3B1*^K700E^ (74 and 49 junctions, respectively, 97 in total) showed coherent overexpression with increased usage of cryptic 3′ss located 10–30nts upstream of the canonical 3′ss (Fig. 3c, d). It is worth noting that even though maximum is least robust statistic, ∆SI_max_ shows amplitude of changes in the “best” reacting replicate and is instrumental in exploratory analysis of marginally small experimental groups.

To recapitulate the homozygous state of *SUGP1* mutants found in tumors, we generated a haploid cell model harboring the *SUGP1*^P636L^ mutation (HAP1^SUGP1-P636L^) by CRISPR/cas9 editing. HAP1^SUGP1-P636L^ cells displayed splice aberrations consistent with the *SF3B1*^mut^ pattern, and mainly characterized by the usage of cryptic 3′ss at 10–25nts upstream the canonical 3′ss (Fig. 3e, Supplementary Fig. S14).

The assessment of *DPH5* aberrant junction expression confirmed the induction of the *SF3B1*^mut^–splice pattern in HAP1^SUGP1-P636L^ as compared to HAP1 (Fig. 3c). Strikingly, siRNA-knockdown of the *SUGP1*^P636L^ further increased the aberrant splice index, implying that *SUGP1* mutations lead to a partial loss of function (hypomorphic mutations) accentuated by the mutant knockdown (Fig. 3f). These experiments elucidated the respective role of reduced *SUGP1* expression by LOH and of hypomorphic mutations found combined in tumors and demonstrated that S*UGP1* phenocopies *SF3B1* 3′ss aberration at the whole transcriptome level.

## Conclusion

Here we introduced a new splice factor recurrently mutated in cancers. Starting from the pan-cancer SF3B1-like 3′ss aberration pattern, we explained almost all cases either by new *SF3B1* mutations outside of the known hotspots or by *SUGP1* alterations. Only two SF3B1-like cases were not associated with either *SF3B1 or SUGP1* alterations and only one of these cases (LAML) had a strong alteration pattern, whereas the SKCM case associated with weak 3′ss aberration pattern could be false positive. Furthermore, we previously described one *SF3B1* wild-type UVM case with such 3′ss pattern, and no *SUGP1* alteration were found retrospectively. Thus, it is likely that other genetic alterations remain to be found in cancers, leading to 3′ss altered usage. Despite the extreme rarity of such cases, it will be important to elucidate these remaining cases as they could unravel important new players in 3′ss selection.

Our work not only confirms the results of Zhang et al. [22] and Liu et al. [32], but also extends them by demonstrating no detectable difference between *SF3B1*^mut^ and *SUGP1*^alt^ splice aberration patterns in cancers and in cellular models. We strengthen that only mutations with LOH are associated with 3′ss aberrant phenotype. In addition, we detected two cases with outlying low expression of *SUGP1* (also with LOH), explaining two more cases with 3′ss aberrations. We further validated the dysfunction of SUGP1 mutants by RNA-seq analyses of original isogenic models. Our data are in line with the previously suggested mechanism of the disruption of SUGP1/SF3B1 interaction induced by the pathologic mutants. We also provide direct experimental evidences supporting that the depletion of *SUGP1* together with expression of a *SUGP1* mutation induce the *SF3B1*^mut^-splice pattern in a cumulative manner, which fits the co-occurrence of LOH and *SUGP1* mutations in tumors. In absence of fully inactivated *SUGP1* animal or cellular models, or tumors reported so far, it is still unclear whether *SUGP1* is an essential gene. However, large depletion of *SUGP1* expression, often combined with *SUPG1* hypomorphic variants, are not only tolerated by tumor cells but likely to be associated with the oncogenic process.

## Materials and methods

### Large-scale in silico screening for *SF3B1*^mut^ splice aberrations in tumors

SBTs were generated based on the RNA-seq fastq files for all samples of the 33 tumor types of the TCGA [23, 24]. The list of 1443 aberrant splice junctions previously reported in *SF3B1*^mut^ tumors was generated (40nts sequences centered on the aberrant 3′ splice site) [5, 6, 21]. Using the SBT structure and the Seven Bridges cloud platform [23, 24], we obtained SBT scores, corresponding to the occurrence of junctions for each tumor sample: each junction was scored as 1 if found and 0 if not (two mismatches were allowed).

A linear model (SBT score ~RNA-seq bam size in gigabytes [GB]) was fit using R custom script excluding top 1% of SBT scores. Fitted values were obtained for all samples and the residuals were taken to characterize adjusted SBT score of the tumors.

*SF3B1* mutations in the 138 cases with the highest SBT scores (cutoff determined by the lowest SBT score of a validated *SF3B1*^A633V^ case) were checked using preprocessed vcf files from the TCGA, samtools mpileup [33] in the wild-type cases, followed by manual inspection using IGV; only expressed *SF3B1* mutations (detectable in RNA-seq) were taken into account and only the 500–800aa region was considered for *SF3B1* mutations.

### RNA-seq preprocessing and selection of junctions

Analysis of splice junctions was performed using STAR 2.5.0 [34] with default parameters (using—quandMode GeneCounts, mapped on hg19 or hg38). Junctions were extracted, annotated as canonical or aberrant based on the gene annotation (NCBI Refseq from UCSC) and normalized using quantiles of canonical junctions (CQ and AQ denote quantiles of canonical and aberrant junctions). Aberrant junctions were paired with the corresponding canonical junctions and annotated as half-canonical 3′ proximal (aberrant junctions ≤100nts to canonical 3′), half-canonical 5′ proximal (aberrant junctions ≤100nts to canonical 5′), half-canonical distant (aberrant junctions >100nts to canonical 3′ or 5′) and aberrant exon (2 exons from the same gene with not annotated junction); junctions with 2 noncanonical ends and chimeric junctions (connecting two different genes) were excluded. Splicing index (SI = N aberrant split reads/(N aberrant + N canonical split reads)) was calculated. For unsupervised aberrant junction selection, we required at least one case from the series to satisfy: (i) SI ≥ SI_min_; (ii) aberrant junction to be expressed AQ ≥ AQ_min_; and (iii) corresponding canonical junction to be expressed CQ ≥ CQ_min_, where SI_min_, AQ_min_, and CQ_min_ are minimal values defined for each series. Junction selection was performed using custom R-script.

### Pan-TCGA analysis of cryptic 3′ splice junctions

RNA-seq data were preprocessed as described above (mapping on the Seven Bridges cloud platform, hg38) on 456 selected cases, including 128 out of the 138 cases with high SBT scores (RNA-seq coverage in ten cases was below the lower limit of 3GB for junction analysis, Supplementary Table 1); and 328 cases with low SBT scores, including 182 cases carrying splice mutations (91 *SF3B1*, 49 *U2AF1*, and 48 *SRSF2*, whatever the position of the mutation—some tumors harboring more than one mutation) and 146 control tumors wild-type for splicing genes from the 21 tumor types having at least one case with high SBT score. Cases with mutations in *U2AF1, SRSF2* and out of hotspot region *SF3B1* were extracted from the vcf files from the TCGA. Proximal 3′ junctions 5–50nts upstream to canonical 3′ss were selected for further analysis if at least one case in the series satisfy the condition SI ≥ 0.15, AQ ≥ 0.4, and CQ ≥ 0.5 (*n* = 975 junctions). To overcome tissue heterogeneity, we also required that the corresponding canonical junctions show at least marginal expression (CQ ≥ 0.3) in 95% of cases. PCA was applied on the set of 336 junctions characterized by SI (R v3.6.1).

### Screening for *SUGP1* mutations in the TCGA

*SUGP1* mutations were extracted from vcf files from the TCGA (exome VAF > 0.1, RNA-seq VAF > 0.3 for missense mutations). Allelic status was determined from SNP-arrays processed by the GAP method [35]. Samtools mpileup [33] was applied to check cases with high SBT scores, followed by manual inspection using IGV of exome and RNA-seq.

### Analysis of junctions of TCGA LUAD

RNA-seq data for LUAD series (514 cases) were preprocessed as described above (hg19). Cases with the lowest RNA-seq coverage (*n* = 75) and cases with *U2AF1* 34F/Y mutations (*n* = 8) or *RBM10* inactivation (*n* = 20) were excluded. Minimal requirements for an aberrant junction to be included were: SI ≥ 0.1, AQ ≥ 0.4, and CQ ≥ 0.5 in at least one case. Half-canonical 3′ proximal (*n* = 5357), half-canonical 5′ proximal (*n* = 1788), half-canonical distant (*n* = 6303) and aberrant exon (*n* = 2501) junctions in *SUGP1* altered (*n* = 5, including mutated and low expression cases), *SF3B1* mutated (*n* = 6) and control (*n* = 400) groups were compared using the bioinformatics protocol described below.

We developed a bioinformatics protocol to test similarity of the global aberrant splicing patterns in *SF3B1*^mut^ and *SUGP1*^alt^ tumors relative to Controls: (i) we applied a Wilcoxon nonparametric rank test to compare SI in *SUGP1*^alt^ vs. Controls and *SF3B1*^mut^ vs. Controls independently, and selected differentially expressed aberrant junctions (*p* value < 0.05, not corrected for multiple testing); (ii) We applied PCA and selected major (with proportion of explained variance >5%) PCs associated with the *SF3B1*^mut^ or *SUGP1*^alt^ groups. We consider PC to be associated to *SF3B1*^mut^ or *SUGP1*^alt^ groups when the PC scores separate mutated cases from the Control group; (iii) If such PCs are found, we considered the junctions correlated with these PCs (Pearson correlation >0.2 or PC loadings >0.05) to be specific to the mutated splicing factor. This protocol was applied to each type of aberrant junctions; intron retention was not considered because of not sufficient RNA-seq coverage in LUAD.

To test significance, the number of shared junctions was compared with the random expected value (obtained using resampling simulation). To refine the set of shared junctions, we re-evaluated the estimation of aberrant expression in the union of *SF3B1*^mut^ and *SUGP1*^alt^ specific aberrant junctions using maximal SI (SI_max_) in the groups. We consider a junction (i) as shared if SI_max_ > SI_Q95_ (95 quantile of the Controls) for both groups; (ii) as definitively not shared if one SI_max_ > SI_Q95_ and the other SI_max_ < SI_Q75_; (iii) as not determined otherwise. Not determined junctions were excluded from further comparison.

### RNA sequencing of HEK293 and HAP1 cell lines

The total RNA was isolated from cells using a NucleoSpin Kit (Macherey-Nagel). cDNA synthesis was conducted with MuLV Reverse Transcriptase in accordance with the manufacturers’ instructions (Invitrogen), with quality assessments conducted on an Agilent 2100 Bioanalyzer. Libraries were constructed using the TruSeq Stranded mRNA Sample Preparation Kit (Illumina) and sequenced on an Illumina HiSeq 2500 platform using a 100-bp paired-end sequencing strategy. The mean number of reads was 134 million, with a mean of 125 million uniquely mapped reads. RNA-seq data analysis was performed as previously described [5].

Paired-end RNA-seq of experimental cell model HEK293T were preprocessed as described above (hg19). Minimal requirements for an aberrant junction to be included were: SI ≥ 0.1, AQ ≥ 0.2, and CQ ≥ 0.5 in at least one case; junctions with CQ < 0.5 or AQ > 0.4 (not expressed canonical or well-expressed aberrant) in the control groups were excluded. Half-canonical 3′ proximal (*n* = 786), half-canonical 5′ proximal (*n* = 412), half-canonical distant (*n* = 1462), and aberrant exon (*n* = 760) junctions were characterized by max(SI) in each group (*SF3B1*^K700E^, *SF3B1*^WT^, SUGP1^KD^, and siContol). To characterize effect of *SUGP1* knockdown and *SF3B1*^K700E^ overexpression, we used relative shift in maximal splice indexes: ∆SI_max_ = (max(SI_Exp_) − max(SI_Control_))/max(SI_Control_)).

Fastq files from paired-end RNA-seq of HAP1 isogenic cell lines were mapped on Human genome (hg19) using STAR (v2.5.3a). BAM files were mined with rMATS tools to detect all alternative splicing events. Junction with False Discovery Rate below 0.01 and |log_2_(SI_Mut_/SI_WT_)| higher than 1 were selected as differentially expressed between WT and *SUGP1*^P636L^ samples.

### Cell culture and transfection

HEK293T and HAP1 cell lines were cultured in DMEM supplemented with 10% fetal bovine serum. A point mutation in *SUGP1* resulting in P636L amino-acid substitution was introduced using CRISPR/cas9-stimulated homology-directed repair to generate isogenic HAP1 cell lines. A second point mutation in *ATP1A1* was simultaneously introduced conferring ouabain resistance [36]. Cells were transfected with preformed Cas9 ribonucleoprotein complexes (RNPs) containing synthetic crRNAs specific for *ATP1A1* and *SUGP1* along with ssODNs-specified mutation at a 3:1 ratio (Integrated DNA Technologies, Inc, IDT). Sequence of gRNA: TGGTACGCATGTCCTCCTGG; donor sequence: TCCTACCACCACCCTCTCCTGTCGACTGAGCAAAGGCAGCCCAGGAGGACATGCGTACCAGGAGGTTGGGCCGGAAGCGGTAGGCCAGCATCATCCTCTTGCGGAACGCCTCATACTCGTCGTCCTCC. Cells were selected and expanded in the presence of ouabain (0.5 µM) and verified by Sanger sequencing.

Plasmid transfections were carried out in cell lines using 500 ng of plasmid construct and Lipofectamine 2000 reagent (Invitrogen) according to the manufacturer’s instructions. For siRNA-mediated knockdown, cells were transfected with 50 nM of siRNA using lipofectamine RNAiMAX (Invitrogen) and the following siRNA: siSUGP1 (Cat.No. s33721, Ambion; and GeneSolution Cat.No. 1027416, Qiagen), or control siRNA (Cat.No. S103650318). Total RNA was extracted with NucleoSpin RNA kit (Macherey-Nagel). The quantity and quality of RNA was determined by spectrophotometry (NanoDrop Technologies). Five hundred nanograms of RNA was used as a template for cDNA synthesis with the High Capacity cDNA Reverse Transcription Kit (Applied Biosystems). Twenty-five nanograms of the synthesized cDNA was used as a template for RT–PCR amplification with specific primers for canonical and cryptic forms of DPH5, DLST and ARMC9 as previously reported [5].

### Expression vectors and minigene constructs

pCDNA3.1-Flag vectors containing wild-type and mutated *SUGP1* were synthesized by Genscript Corporation. Wild-type and mutated *SF3B1*, as well as BP and BP’ mutants of TMEM14C and ENOSF1 constructs were previously described [5].

### Immunoblot analysis

Cells were lysed in radioimmunoprecipitation assay buffer, and proteins were quantified using a BCA Protein Assay (Pierce). Equal amounts were separated on SDS–polyacrylamide gel electrophoresis gels. Proteins were transferred to nitrocellulose membranes followed by immunoblotting with specific primary antibodies for SUGP1 (1:1,000; HPA004890, Sigma), Flag (1:1,000, #3165; Sigma), and β-actin (1:2,000; #5313; Sigma). The membrane was then incubated at room temperature for 1 h with either goat anti-rabbit or goat anti-mouse Odyssey secondary antibodies coupled to a 700 or 800 nm. Immunolabelled proteins were detected using the Odyssey Infrared Imaging System (Li-cor).

## Supplementary information

Supplemental Figures

Supplementary Table

## Data Availability

Sequencing data are available as GSE159304.
