# Supplementary material for: Genetic alterations of SUGP1 mimic mutant-SF3B1 splice pattern in lung adenocarcinoma and other cancers
Source: Oncogene. 2020 Oct 14;40(1):85–96. doi: 10.1038/s41388-020-01507-5 (PMC7790757; doi:10.1038/s41388-020-01507-5)
Supplement: Supplementary file 1 — Supplemental Figures [file 41388_2020_1507_MOESM1_ESM.pdf]

Supp Fig. S1

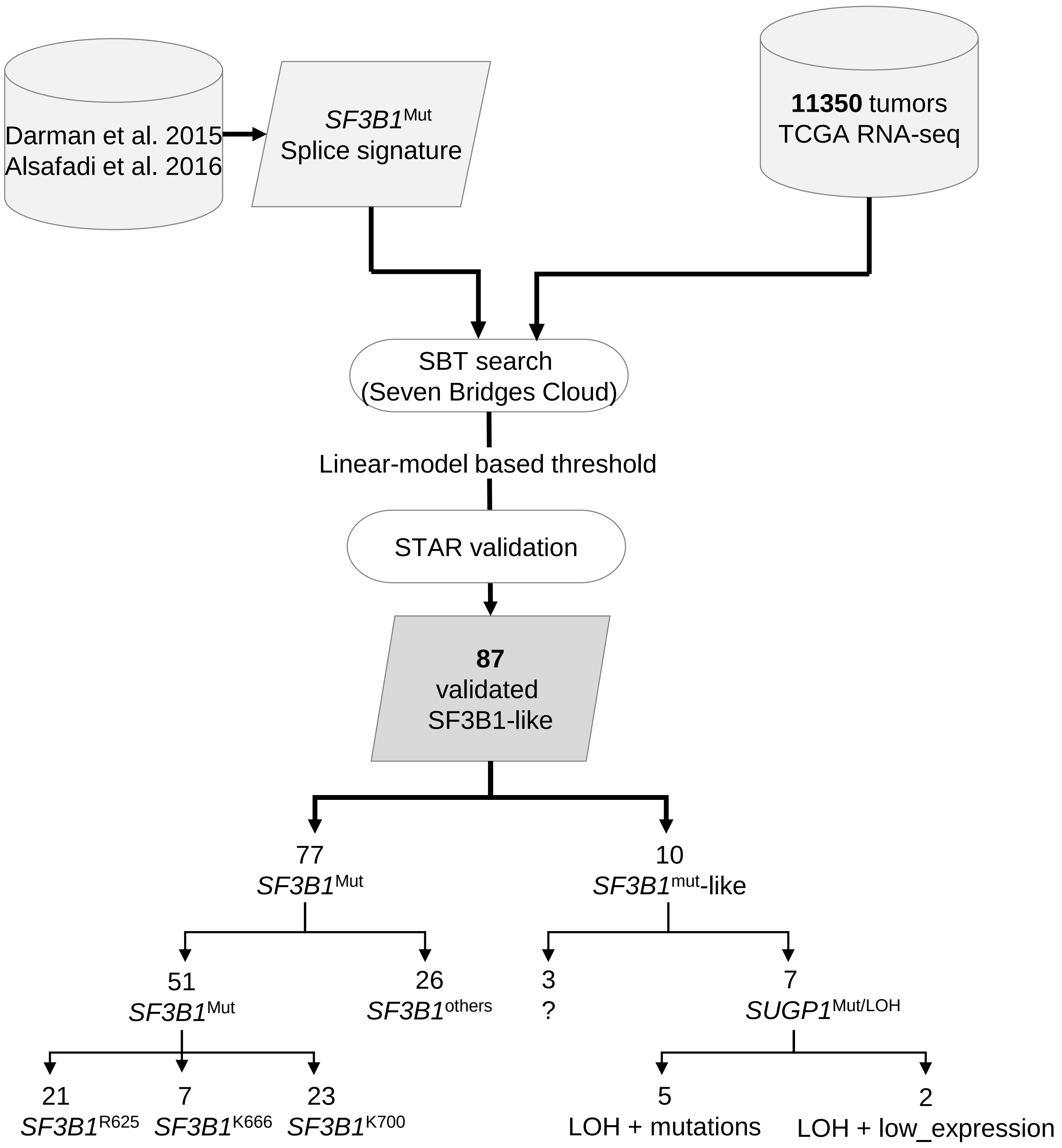

Supplementary figure S1. Workflow of the large-scale detection of *SF3B1*^{Mut} splice pattern through SBT search in 11,350 tumors on Seven Bridges Cloud platform.

Supp Fig. S2

a

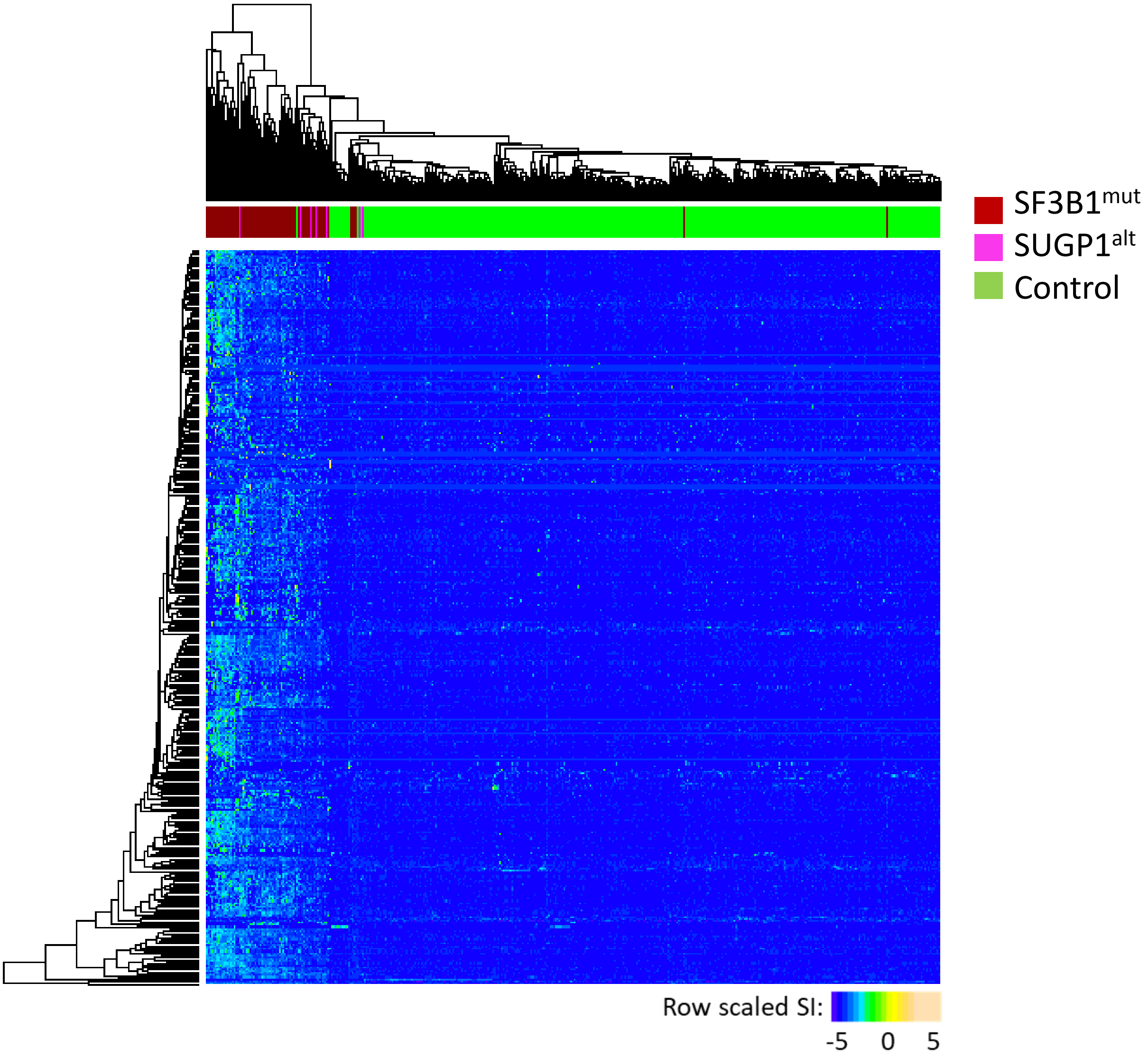

b

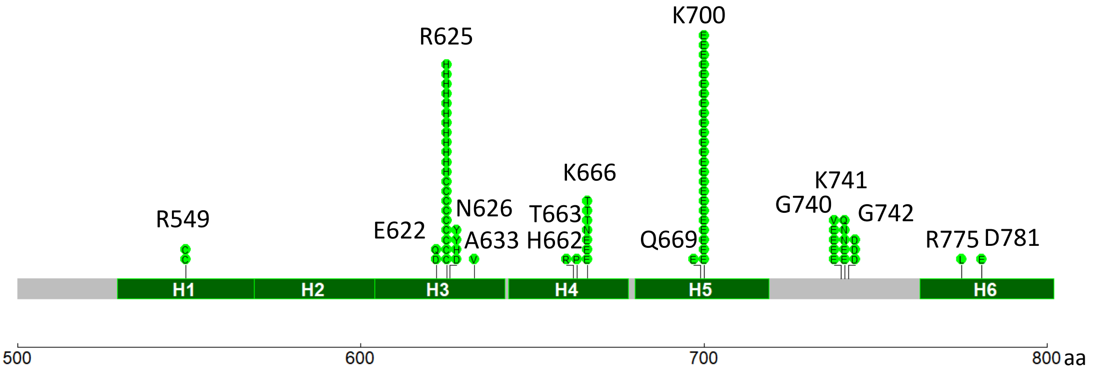

Supplementary figure S2. Hierarchical clustering of SF3B1-like cases detected by SBT and the selection of Control cases by splicing index (SI) of aberrant 3'ss junctions.

a. Junctions (n=365) for clustering were selected based on the PCA analysis shown in (Fig. 1a, right panel): junctions with absolute Pearson correlation more than 0.2 to PC1 were included.

b. Mutations in *SF3B1* found in the TCGA that cause 3'ss aberration. Fragment of SF3B1 500-800aa, containing H1-H6 HEAT domains is shown.

Supp Fig. S3

a

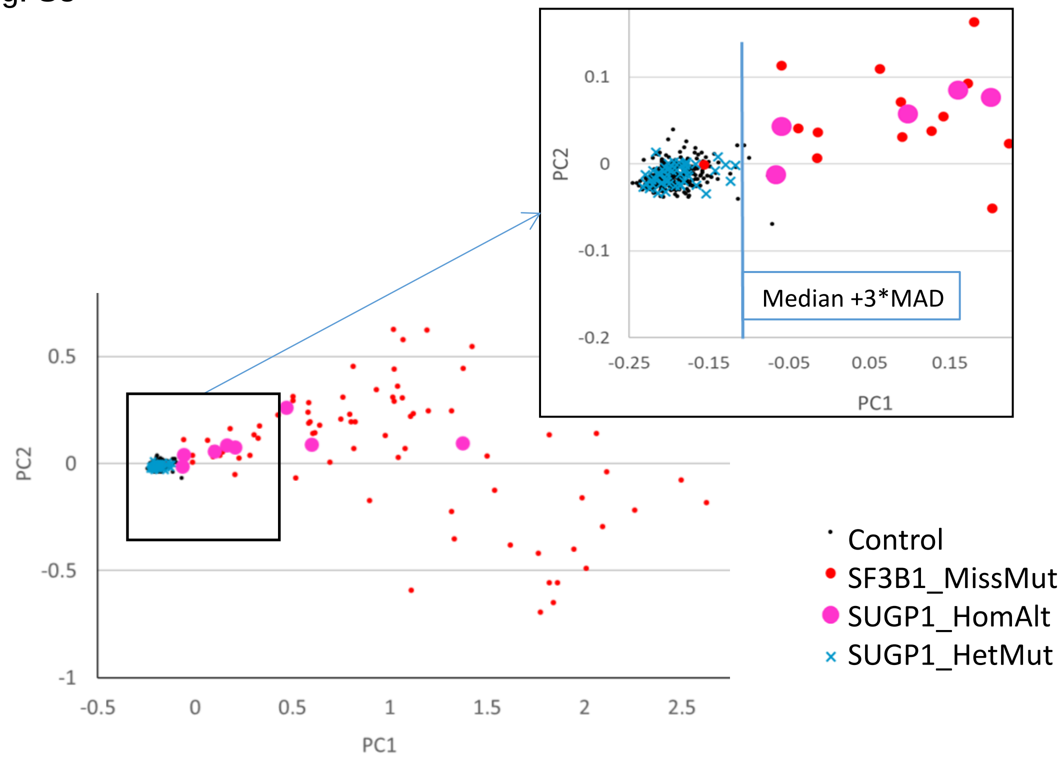

b

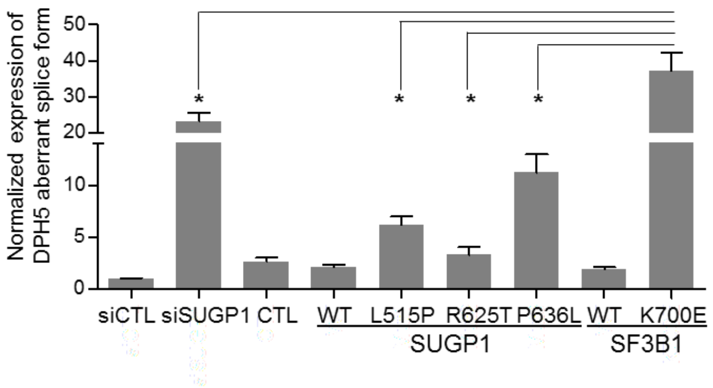

Supplementary figure S3. *SUGP1* mutations in heterozygous state and Fig 1e complement.

a. *SUGP1* mutations in heterozygous state are not associated with SF3B1-like pattern.

Tumors from the TCGA that harbor missense or deleterious mutations of *SUGP1* in heterozygous state were tested using the same approach as in Fig.1b. Principal component analysis was applied to the set of 485 tumors, including 54 cases with *SUGP1* alteration: 8 mutations (7 missense and 1 deleterious) + LOH, 19 deleterious and 27 missense mutations in heterozygous state, characterized by 190 junctions with high loadings for the first principal component in Fig.1b. Principal component analysis showed that all *SUGP1* heterozygous mutations do not exceed Median+3MAD largely determind by the Control cases.

b Effect of siRNA-mediated knockdown of SUGP1, overexpression of wild-type SUGP1 or SF3B1, overexpression of SUGP1-L515P, -R625T or -P636L or SF3B1K700E on the aberrant splice form of DPH5 in HEK293T cell line. Relative expression of cryptic 3’ss junction was determined by quantitative RT–PCR. The results are average of three replicates and are represented as mean±sd, and each condition is compared to the SF3B1-K700E (Paired t-test; *, $p<0.05$).

Supp Fig. S4

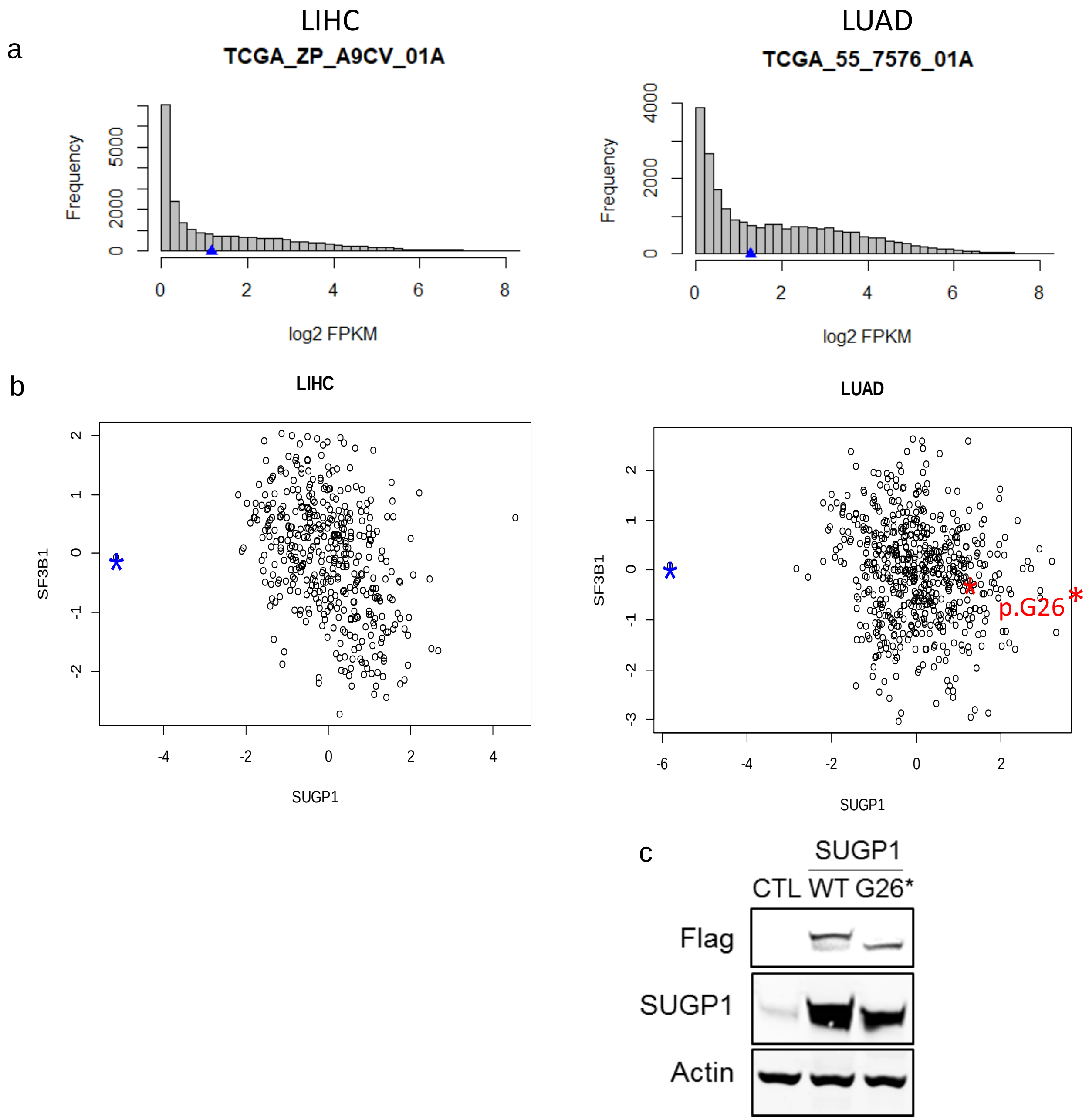

Supplementary figure S4. *SUGP1* expression relative to other genes in a sample (a); normalized *SUGP1/SF3B1* expression in LIHC and LUAD (b); and validation of the G26* stop mutation in SUGP1 leading to an N-terminal-truncated protein (c).

a. Expression of *SUGP1* relative to other genes in the sample (histograms of RNA-seq FPKM for top 24000 genes data; position of *SUGP1* is marked by the blue triangle).

b. b. Normalized RNA-seq FPKM expression of *SUGP1* and *SF3B1* in LIHC (left panel) and LUAD (right panel) cohorts illustrate the lowest *SUGP1* expression (blue star) in the two cases that exhibit SF3B1-like aberrant splice pattern TCGA-ZP-A9CV-01A in LIHC and TCGA-55-7576-01A in LUAD. The red star show the case with stop-gain mutation in *SUGP1* (p.G26*), which displays high expression of the gene.

c. HEK293T cells were transfected with c-terminal-flag expression vectors of wild-type (WT) or G26* mutated SUGP1. The protein overexpression was confirmed by immunoblotting with anti-Flag and anti-SUGP1 using β-actin as a loading control.

Supp Fig. S5

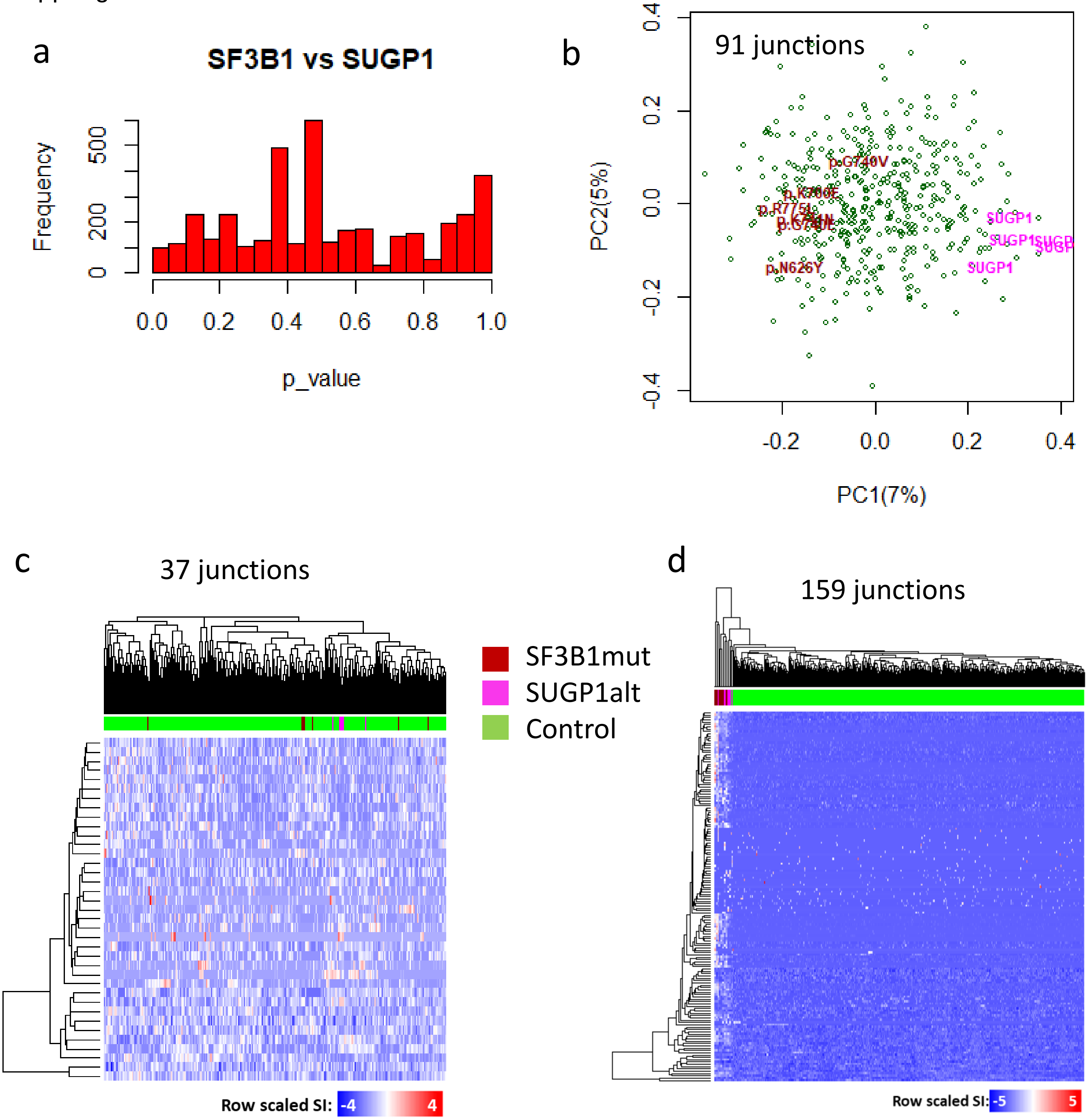

Supplementary figure S5. LUAD 3' ss aberrant junctions in SUGP1^{alt} and SF3B1^{mut}

a. P-value distribution in SUGP1^{alt} vs SF3B1^{mut} comparison in the LUAD series performed by Wilcoxon rank test on the splicing index (SI) of 3' ss aberrant junctions.

b. Principal component 2D plot for the set of aberrant 3' ss junctions with p-value <0.05. SUGP1^{alt} cases and SF3B1^{mut} cases are indicated.

c. Hierarchical clustering of LUAD cases based on 37 junctions with high loadings in PC1.

d. Hierarchical clustering of LUAD cases based on 159 junctions with high loadings in PCAs shown in Fig. 2b for aberrant 3'ss in SUGP1^{alt} vs Controls and SF3B1^{mut} vs Controls comparisons

Supp Fig. S6

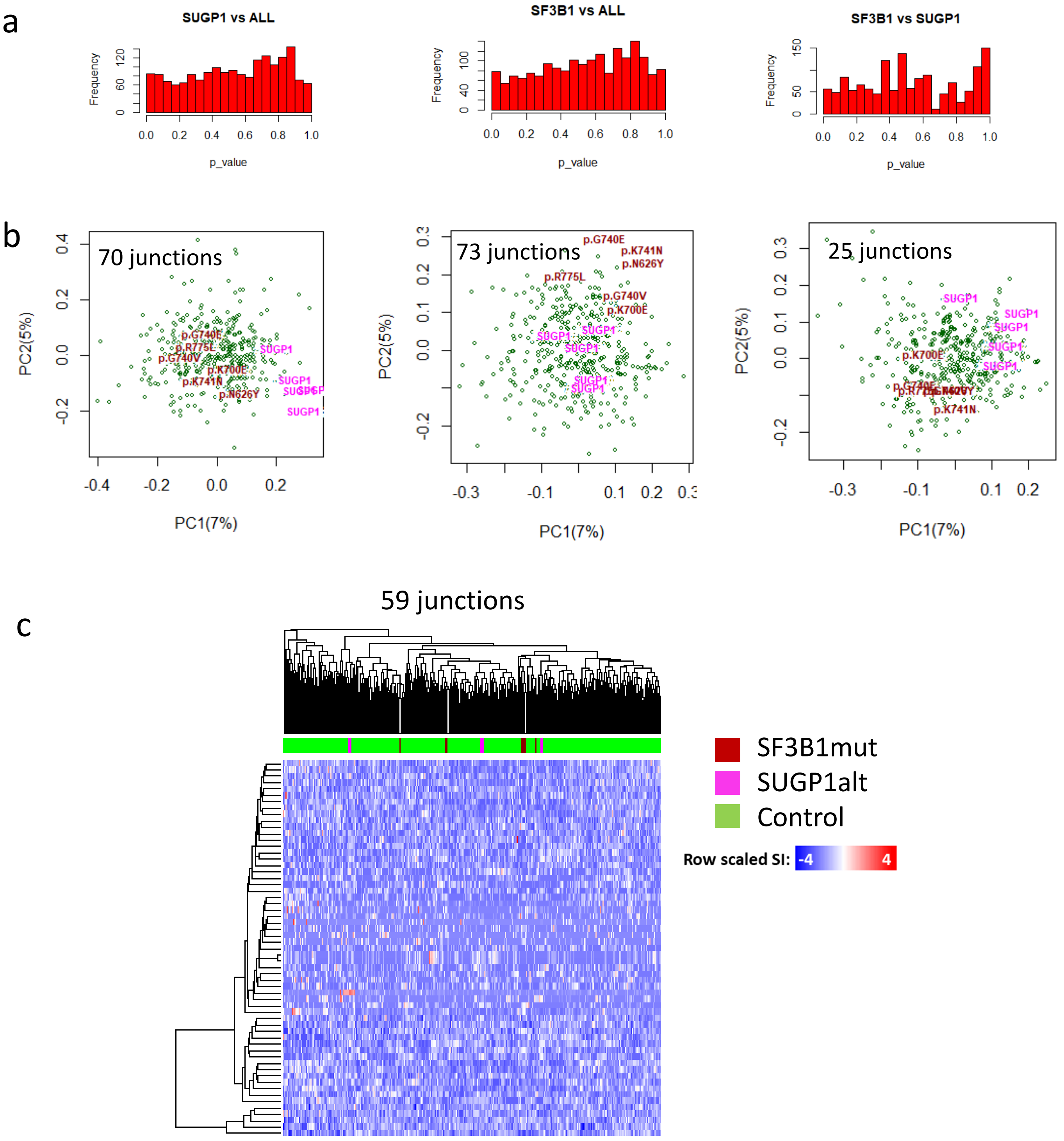

Supplementary figure S6. LUAD 5' ss aberrant junctions in SUGP1^{alt} and SF3B1^{mut}

a. P-value distribution in Wilcoxon rank test of Splice index (SI) in SUGP1^{alt} vs Controls (left panel), SF3B1^{mut} vs Controls (central panel) and SUGP1^{alt} vs SF3B1^{mut} (right panel).

b. Principal component analyses of junctions from the comparison corresponding to a. on the selection of junctions with $p<0.05$.

c. 59 junctions with high loadings from 3 analyses: SUGP1^{alt} cases and SF3B1^{mut} cases are indicated.

Supp Fig. S7

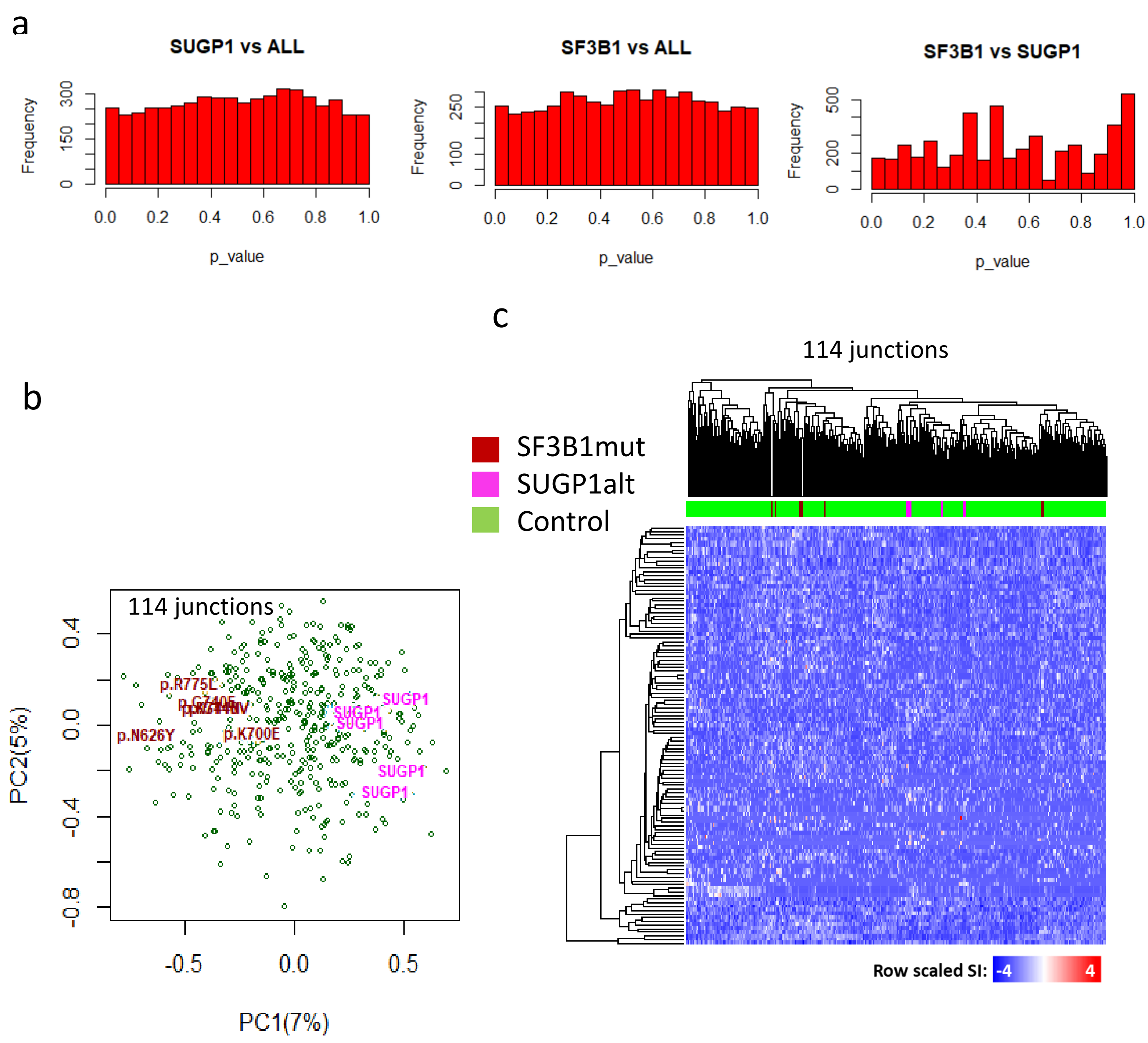

Supplementary figure S7. LUAD 3'&5' ss distant aberrant junctions in SUGP1^{alt} and SF3B1^{mut}

a. P-value distribution in Wilcoxon rank test of Splice index (SI) in SUGP1^{alt} vs Controls (left panel), SF3B1^{mut} vs Controls (central panel) and SUGP1^{alt} vs SF3B1^{mut} (right panel).

b. Principal component analysis on the selection of junctions (n=114) associated to the differential expression (p-value<0.05) extracted from 3 comparisons.

c. Hierarchical clustering on the selection of junctions (n=114) associated to the differential expression extracted from 3 comparisons.

Supp Fig. S8

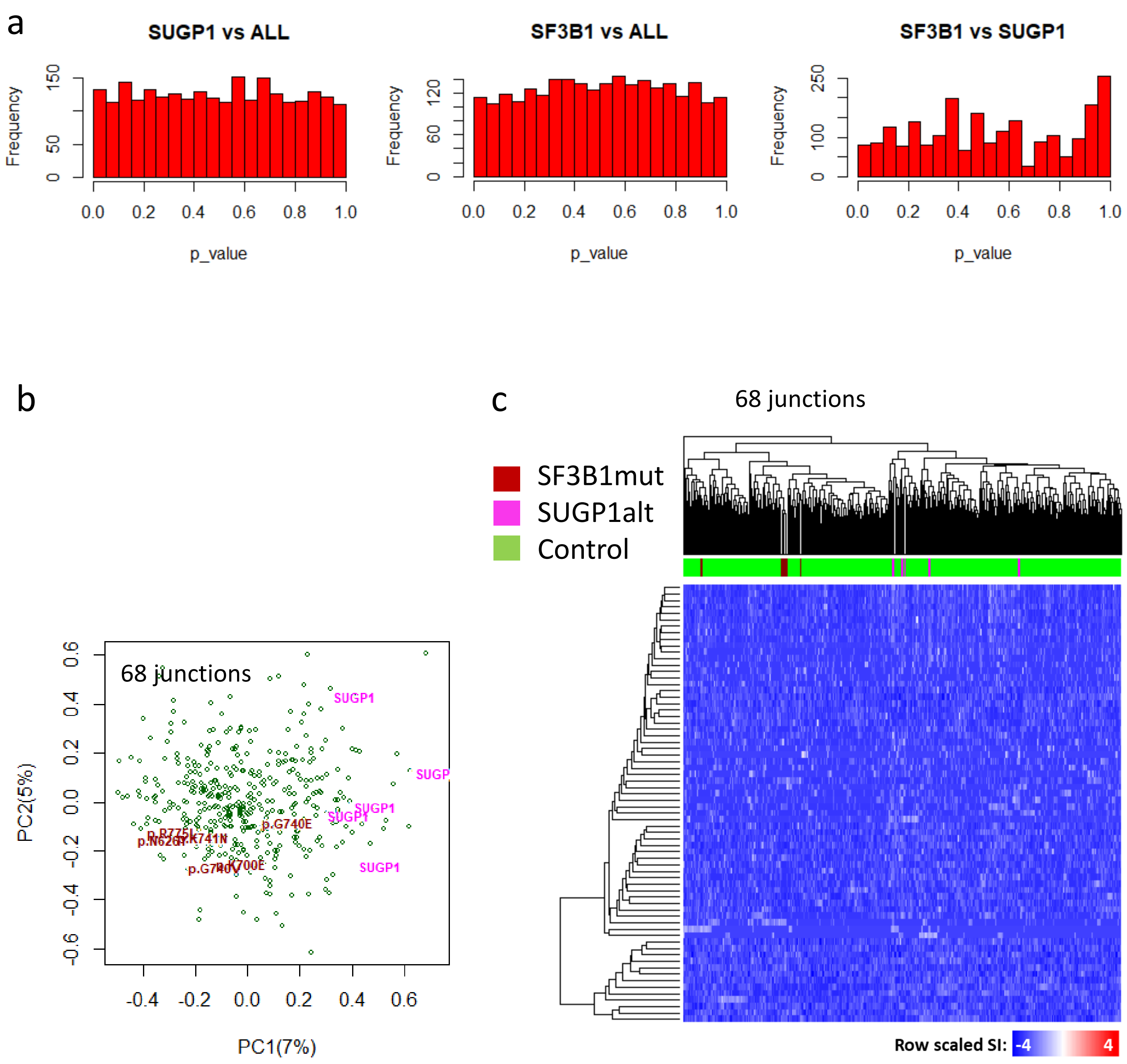

Supplementary figure S8. LUAD exon (exon skipping) aberrant junctions in SUGP1^{alt} and SF3B1^{mut}

a. P-value distribution in Wilcoxon rank test of Splice index (SI) in SUGP1^{alt} vs Controls (left panel), SF3B1^{mut} vs Controls (central panel) and SUGP1^{alt} vs SF3B1^{mut} (right panel).

b. Principal component analysis on the selection of junctions (n=68) associated to the differential expression (p-value<0.05) extracted from 3 comparisons.

c. Hierarchical clustering on the selection of junctions (n=68) associated to the differential expression extracted from 3 comparisons.

Supp Fig. S9

a

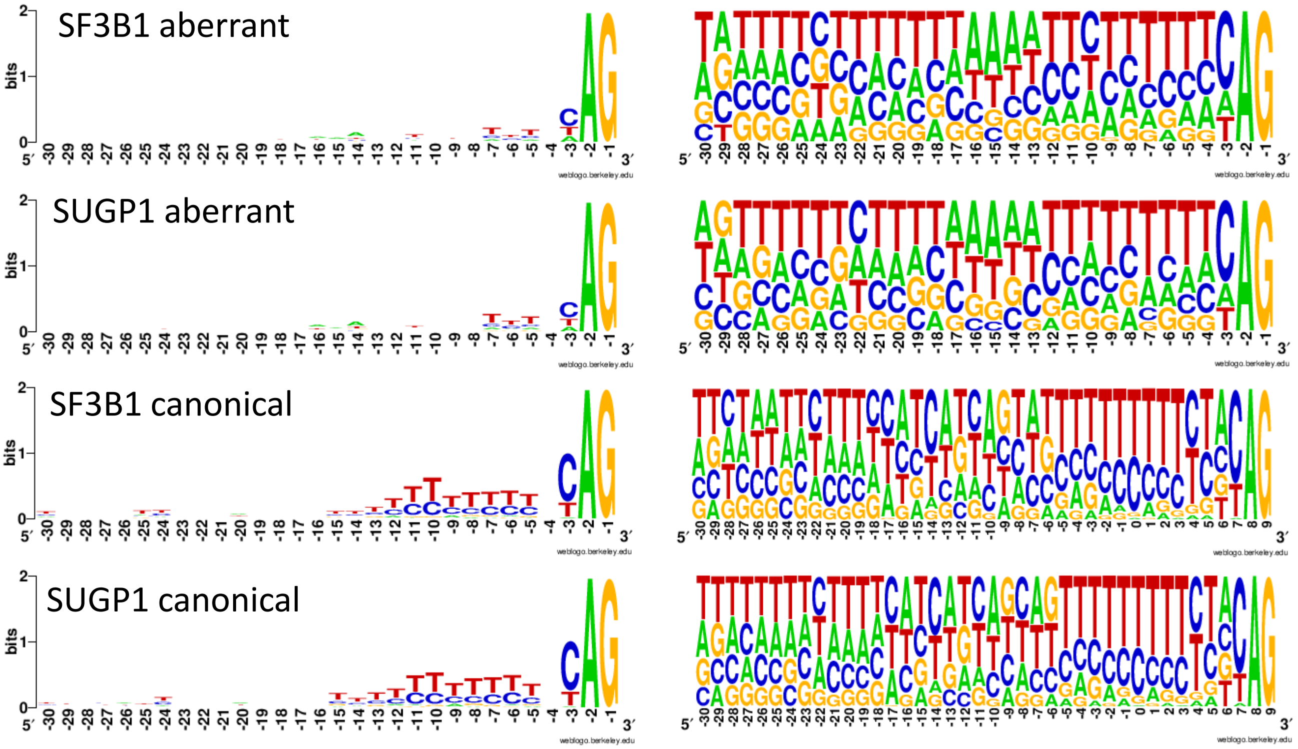

b

Gene described previously	Altered in	Shift, nts
PPP2R5A	both	13
BRD9	SF3B1^{mut}	10

Gene Cancer census 2019	Altered in	Shift, nts
MECOM	both	18
NFE2L2	both	22
NUP98	both	13

c

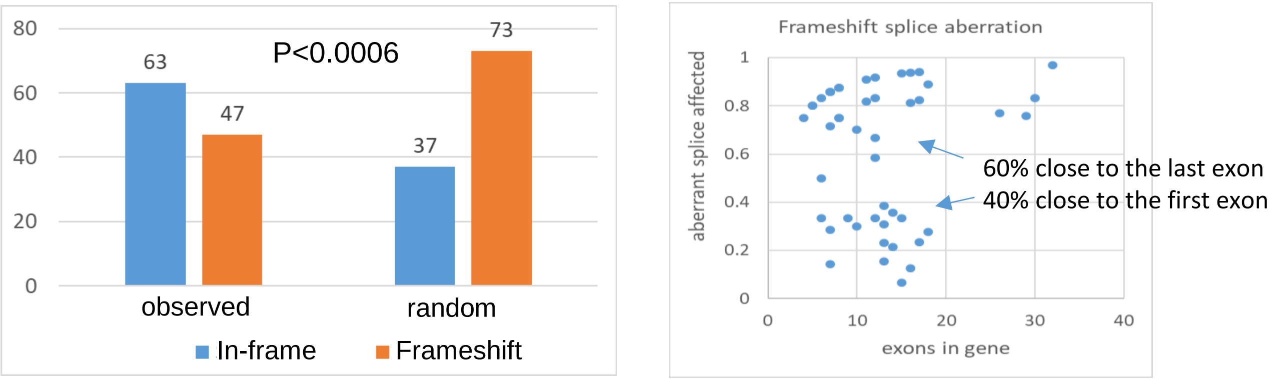

Supplementary figure S9. Aberrant junctions and genes affected in the LUAD cohort

a. Weblogo for nucleotide composition around aberrant and canonical 3’ss.

b. Genes affected by aberrant splicing as compared to the genes described previously and to the Cancer Gene Census 2019 (including >700 genes).

c. Nonsense Mediated Decay (NMD) and 3’ss aberrations observed in LUAD for SF3B1^{mut} and SUGP1^{alt}. Left panel shows overrepresentation of in-frame shifts, evidencing reduced appearance of frameshift shifts probably due to NMD. Right panel shows the exons affected depending on relative position of exon within the gene.

Supp Fig. S10

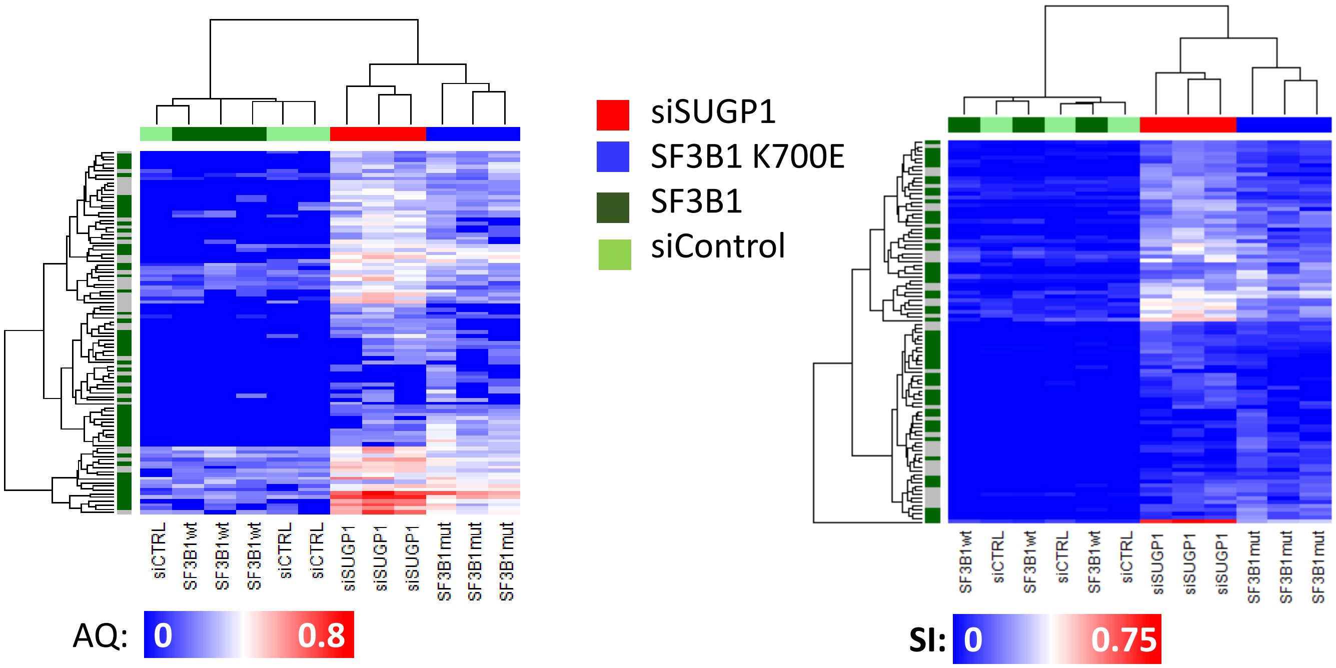

Supplementary figure S10. HEK293T SUGP1^{KD} vs SF3B1^{K700E} models comparison: Aberrant 3'ss usage

Hierarchical clustering and the heatmap of HEK293T models based on aberrant quantile (AQ: left panel) and Splice Index (SI: right panel) of 97 junctions selected from 3'ss aberration comparison with relative splicing index (ΔSImax) >1: SUGP1^{KD} vs siControl (n=74), SF3B1^{K700E} vs Control (n=49), intersection n=26.

Supp Fig. S11

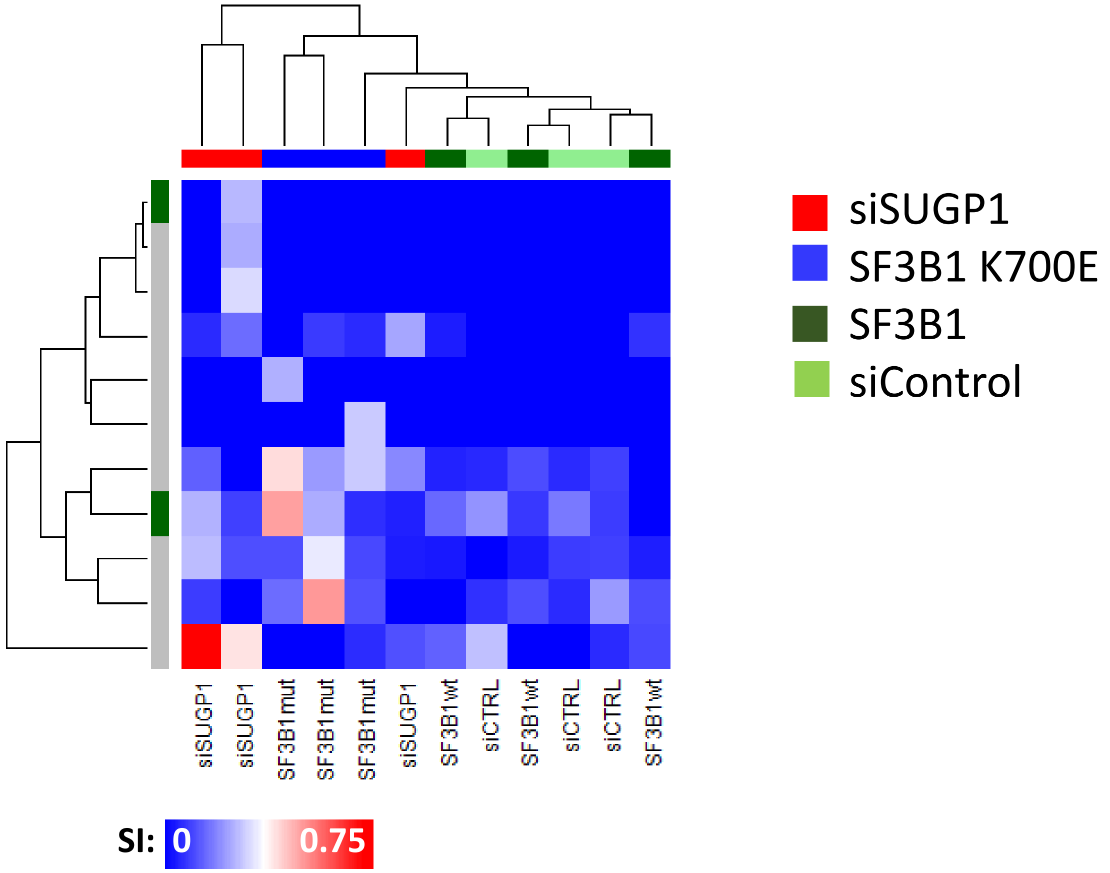

Supplementary figure S11. HEK293T SUGP1^{KD} and SF3B1^{K700E} models comparison: Aberrant 5'ss usage

Hierarchical clustering and the heatmap of HEK293T models based on Splice Index (SI) of 11 junctions selected from 5'ss aberration comparison with relative splicing index ΔSI >1 in *SUGP1*-depleted or K700E overexpressing *SF3B1* HEK293T cells: 412 junctions tested.

Supp Fig. S12

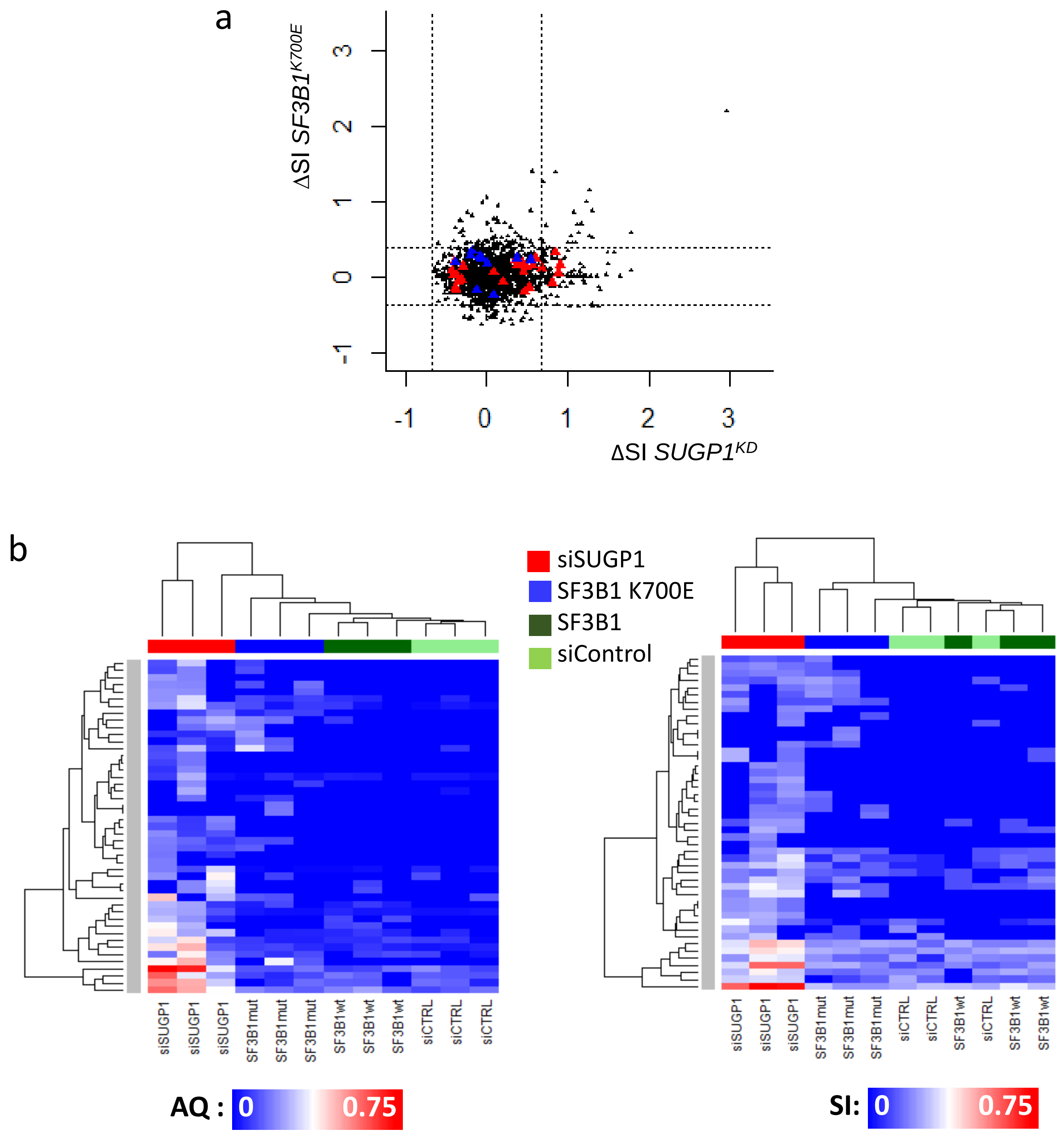

Supplementary figure S12. HEK293T SUGP1^{KD} and SF3B1^{K700E} models comparison: 3'&5'distant (>100nts from canonical ss) aberrant junctions

a. Relative SImax in SUGP1^{KD} and SF3B1^{K700E} in 1462 junctions tested.

b. Hierarchical clustering and the heatmap of Aberrant quantile (AQ) and Splice Index (SI) of 47 junctions with ΔSI > 1 in *SUGP1*-depleted or K700E overexpressing *SF3B1* HEK293T cells.

Supp Fig. S13

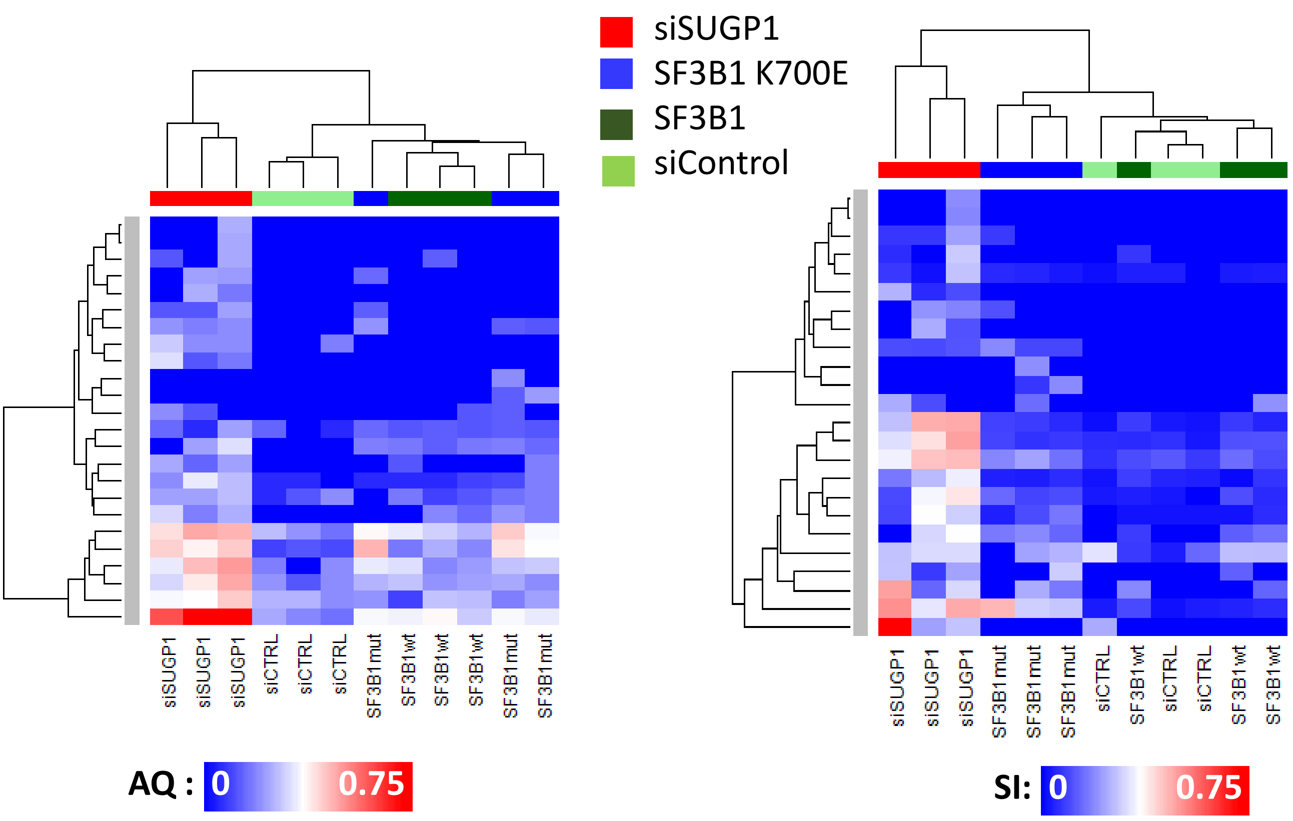

Supplementary figure S13. HEK293T $SUGP1^{KD}$ and $SF3B1^{K700E}$ models comparison: aberrant exon (exon skipping and cassette exon)

Hierarchical clustering and the heatmap of Aberrant quantile (AQ: left panel) and Splice Index (SI: right panel) in 24 junctions with ΔSI > 1 in *SUGP1*-depleted or K700E overexpressing *SF3B1* HEK293T cells: 760 junctions tested.

Supp Fig. S14

a

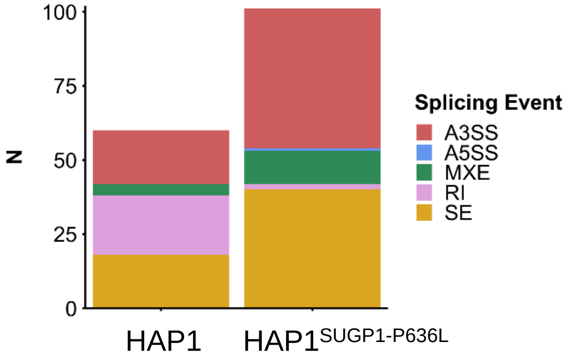

b

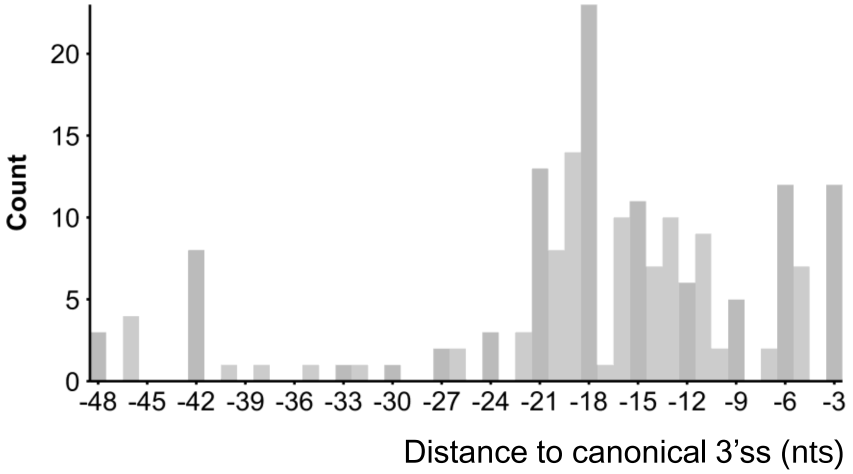

Supplementary figure S14 SF3B1^{MHS}-like splice pattern analysis in HAP1$^{SUGP1\text{-}P636L}$ isogenic cell line

a. Barplot representing the expressed aberrant splicing events in HAP1 and HAP1$^{SUGP1\text{-}P636L}$. A3SS: junctions with alternative 3' splice site; A5SS: junctions with alternative 5' splice site; MXE: junctions with alternative 3'SS and 5'SS; RI: intron retention; SE: exon skipping.

b. Distances between the cryptic and canonical 3'ss in the top differentially expressed junctions in HAP1 and HAP1$^{SUGP1\text{-}P636L}$ isogenic cell lines. Negative distances mean the cryptic 3'ss is upstream of the canonical 3'ss. The 0 point marks the position of the canonical 3'ss.
