# Supplementary material for: Genetic alterations of SUGP1 mimic mutant-SF3B1 splice pattern in lung adenocarcinoma and other cancers
Source: Oncogene. 2020 Oct 14;40(1):85–96. doi: 10.1038/s41388-020-01507-5 (PMC7790757; doi:10.1038/s41388-020-01507-5)
Supplement: Supplementary file 2 — Supplementary Table [file 41388_2020_1507_MOESM2_ESM.pdf]

Supplementary Table 1. List of tumor samples associated with high SBT scores and controls tested by STAR

	Status_STARj	Sample_ID	Cohort	SBT_seq_found	Corrected_S	SBT_size_corrected_Score	RNAseq_size	PC1_44%	Cutoff_PC1_N	PC2_6%	Splice_pattern	SUGP1_status	SF3B1_mutatic	Comment	Causative_mutation	SRSF2_mutation_	U2AF1_mutatior	Tumor_content	SF3B1mut_VAF_WXS	SF3B1mut_VAF_RNAseq
1	tested	TCGA-WC-AA9E-01A	UVM	889	801	800,07	7,3	1,88395013		0,13938282	SF3B1_like		p.R625H		SF3B1_p.R625H			1	0,46	
2	tested	TCGA-VD-A8KH-01A	UVM	933	795	810,04	9,2	2,28820914		-0,120655	SF3B1_like		p.R625H		SF3B1_p.R625H			1	0,42	
3	tested	TCGA-VD-A8K9-01A	UVM	906	789	797,82	8,4	1,75133848		-0,1325874	SF3B1_like		p.R625C		SF3B1_p.R625C			1	0,53	
4	tested	TCGA-V4-A9EW-01A	UVM	864	760	764,39	7,9	1,85301063		0,14205208	SF3B1_like		p.R625C		SF3B1_p.R625C			0,7	0,43	
5	tested	TCGA-VD-A8KA-01B	UVM	821	725	726,24	7,6	1,93720817		0,13207115	SF3B1_like		p.R625C		SF3B1_p.R625C			1	0,55	
6	tested	TCGA-DK-A1AE-01A	BLCA	908	717	751,26	11,2	1,44375237		0,05157389	SF3B1_like		p.R625C		SF3B1_p.R625C			0,83	0,39	
7	tested	TCGA-FR-A7U8-06A	SKCM	774	715	703,03	6,2	1,97498466		0,09845813	SF3B1_like		p.R625H		SF3B1_p.R625H			0,95	0,43	
8	tested	TCGA-V4-A9F4-01A	UVM	758	704	691,55	6	1,67563238		0,0904407	SF3B1_like		p.R625H		SF3B1_p.R625H			0,7	0,42	
9	tested	TCGA-RP-A690-06A	SKCM	806	702	705,88	7,9	1,81567809		-0,128992	SF3B1_like		p.R625H		SF3B1_p.R625H			0,95	0,46	
10	tested	TCGA-V4-A9ES-01A	UVM	817	676	693,08	9,3	2,26670959		-0,0167202	SF3B1_like		p.R625H		SF3B1_p.R625H			1	0,35	
11	tested	TCGA-25-1631-01A	OV	935	646	713,23	14,9	1,46370255		0,01809278	SF3B1_like		p.K700E		SF3B1_p.K700E			1	NA	
12	tested	TCGA-V4-A9EJ-01A	UVM	757	640	647,77	8,4	1,7679293		0,24031942	SF3B1_like		p.R625H		SF3B1_p.R625H			1	0,41	
13	tested	TCGA-EW-A1P5-01A	BRCA	698	623	616,59	6,8	1,21393961		0,04059848	SF3B1_like		p.K700E		SF3B1_p.K700E			0,64	0,65	
14	tested	TCGA-ER-A3ES-06A	SKCM	597	622	582,08	3	1,78332544		-0,0393472	SF3B1_like		p.R625H		SF3B1_p.R625H			0,95	0,7	
15	tested	TCGA-VD-A8KB-01A	UVM	700	617	614,32	7,1	1,69380612		0,02680319	SF3B1_like		p.R625C		SF3B1_p.R625C			1	0,4	
16	tested	TCGA-GN-A4U4-06A	SKCM	728	611	619,85	8,4	1,07883193		-0,0445803	SF3B1_like		p.N626Y		SF3B1_p.N626Y			0,83	0,28	
17	tested	TCGA-A2-A0EV-01A	BRCA	677	610	602,39	6,5	1,06438029		0,20109154	SF3B1_like		p.K700E		SF3B1_p.K700E			0,6	0,44	
18	tested	TCGA-55-7576-01A	LUAD	823	605	647,99	12,2	1,19034853		0,07575339	SF3B1_like	LowestExp_LOH			SUGP1_theLowestExpr_LOH			0,7		
19	tested	TCGA-ER-A2NF-01A	SKCM	809	605	657,36	11,7	1,43580404		0,30586875	SF3B1_like		p.R625H		SF3B1_p.R625H			0,95	0,31	
20	tested	TCGA-C8-A26V-01A	BRCA	840	596	648,49	13,2	0,92623745		0,04685319	SF3B1_like		p.K700E		SF3B1_p.K700E			0,93	0,29	
21	tested	TCGA-BH-A18M-01A	BRCA	659	568	576,77	7,4	0,77390987		-0,0382578	SF3B1_like		p.K700E		SF3B1_p.K700E			0,45	0,27	
22	tested	TCGA-AC-A2B8-01A	BRCA	732	562	588,33	10,4	0,58040956		-0,0187185	SF3B1_like		p.E622Q		SF3B1_p.E622Q			0,59	0,36	
23	tested	TCGA-AN-A0XO-01A	BRCA	697	543	565,17	9,8	0,91830986		0,09092496	SF3B1_like		p.K700E		SF3B1_p.K700E			0,76	0,39	
24	tested	TCGA-DX-AB2J-01A	SARC	686	540	558,92	9,5	1,27411723		-0,1532167	SF3B1_like		p.K700E		SF3B1_p.K700E			0,96	0,42	
25	tested	TCGA-AR-A1AM-01A	BRCA	653	538	547,05	8,3	0,86535362		-0,0123932	SF3B1_like		p.K700E		SF3B1_p.K700E			0,46	0,21	
26	tested	TCGA-VS-A9UQ-01A	CESC	565	517	501,55	5,8	0,94838507		-0,1532775	SF3B1_like		p.K666E		SF3B1_p.K666E			0,78	0,26	
27	tested	TCGA-WC-A881-01A	UVM	556	510	494,20	5,7	1,83433402		-0,3226952	SF3B1_like		p.H662R		SF3B1_p.H662R			1	0,51	
28	tested	TCGA-AB-2929-03A	LAML	609	500	506,33	8,1	1,36203029		-0,6457247	SF3B1_like		p.K700E		SF3B1_p.K700E			1	0,51	
29	tested	TCGA-AB-2944-03A	LAML	575	487	485,85	7,3	1,36868245		-0,6995978	SF3B1_like		p.K700E	new_SF3B1_found	SF3B1_p.K700E			1	NA	
30	tested	TCGA-BH-A201-01A	BRCA	626	485	501,29	9,3	0,64539119		0,07635209	SF3B1_like		p.K700E		SF3B1_p.K700E			0,68	0,23	
31	tested	TCGA-E2-A10F-01A	BRCA	564	481	477,34	7,1	0,56001512		-0,0206506	SF3B1_like		p.D781E		SF3B1_p.D781E			0,8	NA	
32	tested	TCGA-V4-A9E9-01A	UVM	535	479	466,68	6,1	1,64947186		0,41792027	SF3B1_like		p.R625H		SF3B1_p.R625H			1	0,38	
33	tested	TCGA-ZF-A9R4-01A	BLCA	520	469	455,77	5,9	0,60228981		-0,2647282	SF3B1_like		p.G740E		SF3B1_p.G740E			0,92	0,36	
34	tested	TCGA-W5-AA2U-01A	CHOL	535	465	465,68	6,6	0,76184866		-0,1285748	SF3B1_like		p.G740E		SF3B1_p.G740E			1	0,31	
35	tested	TCGA-E9-A1R3-01A	BRCA	673	455	499,42	12,2	0,69888007		-0,1243148	SF3B1_like		p.N626D		SF3B1_p.N626D			0,82	0,26	
36	tested	TCGA-WC-A885-01A	UVM	449	453	420,25	3,8	1,82072792		0,49133154	SF3B1_like		p.R625H		SF3B1_p.R625H			1	0,42	
37	tested	TCGA-AB-2882-03A	LAML	560	443	452,31	8,4	0,75457343		-0,4617783	SF3B1_like			noSF3B1mut_U2AF1_34_S/Y	NA_SF3B1_like		34_S/Y	1		
38	tested	TCGA-BH-A0DQ-01A	BRCA	491	437	431,37	6	0,67300553		0,07489529	SF3B1_like		p.K700E		SF3B1_p.K700E			0,73	0,4	
39	tested	TCGA-13-1408-01A	OV	844	436	544,98	19,4	0,8321929		-0,2936829	SF3B1_like		p.K700E	new_SF3B1_found	SF3B1_p.K700E			0,7	NA	
40	tested	TCGA-86-8281-01A	LUAD	591	432	455,14	10	0,78655951		0,01930276	SF3B1_like		p.N626Y		SF3B1_p.N626Y			0,62	0,29	
41	tested	TCGA-BH-A0HP-01A	BRCA	546	426	435,58	8,5	0,59455823		0,10446298	SF3B1_like		p.K666E		SF3B1_p.K666E			0,59	0,29	
42	tested	TCGA-2A-A8VX-01A	PRAD	474	410	400,43	6,4	1,10615879		-0,3148112	SF3B1_like		p.K700E		SF3B1_p.K700E			0,93	0,48	
43	tested	TCGA-FR-A8YD-06A	SKCM	471	404	394,93	6,5	0,88487248		0,34359793	SF3B1_like		p.K700E		SF3B1_p.K700E			0,86	0,3	
44	tested	TCGA-WC-AA9A-01A	UVM	550	404	422,28	9,5	1,29137195		-0,3108206	SF3B1_like		p.K666T		SF3B1_p.K666T			1	0,43	
45	tested	TCGA-B6-A0WT-01A	BRCA	443	402	384,21	5,5	0,8056956		0,11690236	SF3B1_like		p.K700E		SF3B1_p.K700E			0,87	0,36	
46	tested	TCGA-YZ-A985-01A	UVM	407	398	370,48	4,3	1,57811507		0,29675721	SF3B1_like		p.R625C		SF3B1_p.R625C			1	0,49	
47	tested	TCGA-IB-A7LX-01A	PAAD	486	390	391,54	7,6	0,59299755		-0,279468	SF3B1_like		p.G740E		SF3B1_p.G740E			0,72	0,24	
48	tested	TCGA-DA-A3F8-06A	SKCM	539	388	408,16	9,7	0,57133753		0,03382364	SF3B1_like		p.R625C		SF3B1_p.R625C			0,24	0,18	
49	tested	TCGA-V4-A9EZ-01A	UVM	413	380	360,50	5,2	1,82540486		0,43758414	SF3B1_like		p.R625H		SF3B1_p.R625H			1	0,41	
50	tested	TCGA-J4-A83L-01A	PRAD	497	372	382,90	8,7	0,48443958		-0,2639085	SF3B1_like		p.E622D		SF3B1_p.E622D			0,5	0,13	
51	tested	TCGA-86-8669-01A	LUAD	379	370	341,65	4,3	0,5559819		-0,3829124	SF3B1_like		p.G740E		SF3B1_p.G740E			0,82	0,19	
52	tested	TCGA-V4-A9EA-01A	UVM	359	353	323,51	4,2	1,17686961		0,40630553	SF3B1_like		p.R625C		SF3B1_p.R625C			1	0,41	
53	tested	TCGA-ER-A19S-06A	SKCM	477	352	362,58	8,7	0,3826239		-0,2940908	SF3B1_like		p.R625C		SF3B1_p.R625C			0,24	0,1	
54	tested	TCGA-E2-A56Z-01A	BRCA	438	334	338,44	7,9	0,88772863		0,12366244	SF3B1_like		p.K700E		SF3B1_p.K700E			0,9	0,14	
55	tested	TCGA-ZB-A96F-01A	THYM	400	333	325,38	6,5	0,47666994		-0,0760639	SF3B1_like		p.K700E		SF3B1_p.K700E			0,3	0,24	
56	tested	TCGA-3H-AB3K-01A	MESO	368	325	307,52	5,6	0,73717609		-0,1026744	SF3B1_like	p.R625T_LOH			SUGP1_p.R625T_LOH			1		
57	tested	TCGA-V4-A9E5-01A	UVM	347	322	299,61	4,9	0,88442813		0,35671315	SF3B1_like		p.K666T		SF3B1_p.K666T			1	0,47	
58	tested	TCGA-EP-A26S-01A	LIHC	414	321	330,15	7,5	0,52801318		0,07167389	SF3B1_like		p.K666T		SF3B1_p.K666T			0,96	0,47	
59	tested	TCGA-DV-A4W0-01A	KIRC	413	314	325,93	7,7	0,4845735		-0,2809894	SF3B1_like		p.K741E		SF3B1_p.K741E			0,73	0,37	
60	tested	TCGA-XM-A8RI-01A	THYM	384	291	290,80	7,5	0,58253819		-0,3961074	SF3B1_like		p.K700E		SF3B1_p.K700E			0,3	0,08	
61	tested	TCGA-VP-A87C-01A	PRAD	374	286	283,86	7,3	0,2863288		-0,2154153	SF3B1_like		p.K741E		SF3B1_p.K741E			0,4	0,3	
62	tested	TCGA-V4-A9EC-01A	UVM	308	270	251,12	5,4	1,23964815		0,17263589	SF3B1_like		p.T663P		SF3B1_p.T663P			0,8	0,43	
63	tested	TCGA-BH-A5J0-01A	BRCA	363	264	267,56	7,7	0,47617307		0,22761058	SF3B1_like		p.K700E		SF3B1_p.K700E			0,76	0,15	
64	tested	TCGA-44-2659-01A	LUAD	419	254	279,06	10,2	0,35306395		-0,0080302	SF3B1_like		p.K741N		SF3B1_p.K741N			0,8	0,38	
65	tested	TCGA-99-8025-01A	LUAD	384	238	256,08	9,5	0,23086705		-0,2217776	SF3B1_like		p.G740V		SF3B1_p.G740V			0,8	0,26	
66	tested	TCGA-75-5125-01A	LUAD	257	224	203,67	5,2	0,10982628		-0,0188267	SF3B1_like		p.K700E	new_SF3B1_found	SF3B1_p.K700E			0,6	0,11	
67	tested	TCGA-G8-6324-01A	DLBC	283	198	194,85	7,2	0,48138405		-0,1822521	SF3B1_like		p.K700E	new_SF3B1_found	SF3B1_p.K700E			0,6	0,32	
68	tested	TCGA-05-4432-01A	LUAD	426	195	243,07	12,7	0,28913709		0,03743564	SF3B1_like	p.L515P_LOH			SUGP1_p.L515P_LOH			0,5		
69	tested	TCGA-LD-A74U-01A	BRCA	311	194	202,15	8,4	0,07674131		-0,2649776	SF3B1_like		p.K666E		SF3B1_p.K666E			0,3	0,15	0,2
70	tested	TCGA-B5-A3FA-01A	UCEC	228	193	172,74	5,3	0,09608585		-0,1665763	SF3B1_like		p.R549C	new_causative_SF3B1_mutation	SF3B1_p.R549C			0,93	0,23	
71	tested	TCGA-UY-A78N-01A	BLCA	301	186	194,13	8,3	0,0986739		0,02092703	SF3B1_like		p.Q699E		SF3B1_p.Q699E			0,93	0,76	
72	tested	TCGA-ZP-A9CV-01A	LIHC	207	172	151,70	5,3	0,22953485		0,20827068	SF3B1_like	LowestExp_LOH			SUGP1_theLowestExpr_LOH			0,76		
73	tested	TCGA-3K-AAZ8-01A	LIHC	227	168	157,44	6,2	0,12852082		0,11392518	SF3B1_like		p.N626H		SF3B1_p.N626H			0,53	0,11	0,05
74	tested	TCGA-NJ-A55O-01A	LUAD	217	166	151,94	5,9	0,09064536		-0,1979242	SF3B1_like		p.R775L		SF3B1_p.R775L			0,1	0,1	
75	tested	TCGA-EE-A2A1-06A	SKCM	400	151	204,98	13,4	0,00830143		0,21771868	SF3B1_like			noSF3B1mut	NA_SF3B1_like			0,41		
76	tested	TCGA-NJ-A4YQ-01A	LUAD	215	148	139,53	6,5	0,18183594		-0,3808824	SF3B1_like	p.P636L_LOH			SUGP1_p.P636L_LOH			0,3		
78	tested	TCGA-DD-AAD2-01A	LIHC	167	145	120,74	4,8	0,26639742		0,23102408	SF3B1_like		p.K741N		SF3B1_p.K741N			0,54	0,19	0,03
79	tested	TCGA-AB-2973-03A	LAML	224	125	128,61	7,7	0,59292373		-0,1533019	SF3B1_like		p.K666N		SF3B1_p.K666N			NA	0,4	
80	tested	TCGA-78-7542-01A	LUAD	267	121	140,18	9,5	-0,0013918		-0,0744241	SF3B1_like	p.G519V_LOH			SUGP1_p.G519V_LOH			0,8		
81	tested	TCGA-B0-5698-01A	KIRC	296	118	148,56	10,7	0,17390124		0,03353072	SF3B1_like		p.G742D		SF3B1_p.G742D			0,9	0,2	0,15
82	tested	TCGA-91-6829-01A	LUAD	162	114	105,60	5,8	-0,0168172		0,14361644	SF3B1_like	p.G26*_LOH			SUGP1_p.G26*_LOH_geneExpressedWell			0,8		
83	tested	TCGA-TS-A7P8-01A	MESO	177	107	99,44	6,6	-0,0095529		-0,199582	SF3B1_like		p.G742D		SF3B1_p.G742D			lowTumorContent	0,27	0,15
84	tested	TCGA-MI-A75I-01A	LIHC	184	106	100,99	6,9	0,1252046		0,12493743	SF3B1_like		p.K741Q		SF3B1_p.K741Q			0,88	0,36	
85	tested	TCGA-D8-A1JH-01A	BRCA	344	148	183,83	11,4	-0,0365063	below	-0,1448297	SBT_SF3B1_like		p.K700E	new_SF3B1_found	SF3B1_p.K700E			0,1	0,05	
86	tested	TCGA-CZ-4864-01A	KIRC	262	111	142,64	9,7	-0,0458641	below	-0,152604	SBT_SF3B1_like		p.G742D	new_SF3B1_found	SF3B1_p.G742D			0,7	0,04	0,1
87	tested	TCGA-WC-A87W-01A	UVM	321	233	231,29	7,3	-0,0613243	below	-0,0797157	SBT_SF3B1_like				NA_SF3B1_like			1		
88	tested	TCGA-B6-A0I2-01A	BRCA	171	107	97,00	6,4	-0,093088	below	-0,0742772	SBT_SF3B1_like		p.A633V		SF3B1_p.A633V			0,62	0,55	
89	tested	TCGA-20-1683-01A	OV	677	327	416,90	17,2	-0,0876647	below	0,09655531	Not_Validated_STAR							0,85		
90	tested	TCGA-B7-A5TJ-01A	STAD	509	223	290,52	14,8	-0,1045013	below	0,08268098	Not_Validated_STAR							0,74		

91	tested	TCGA-AK-3454-01A	KIRC	164	163	132,26	4	-0,2447964	below	-0,5675752	Not_Validated_STAR							1		
92	tested	TCGA-A2-A0T3-01A	BRCA	236	148	147,09	7,3	-0,1299961	below	0,14065323	Not_Validated_STAR							0,76		
93	tested	TCGA-25-1312-01A	OV	314	126	158,63	11,1	-0,1534132	below	-0,1495424	Not_Validated_STAR							1		
94	tested	TCGA-GI-A2C9-01A	BRCA	183	119	116,91	6,4	-0,1534796	below	0,06529939	Not_Validated_STAR							0,92		
95	tested	TCGA-GM-A5PV-01A	BRCA	172	113	101,04	6,2	-0,0745518	below	0,04143208	Not_Validated_STAR							0,73		
96	tested	TCGA-C5-A2M2-01A	CESC	172	110	100,36	6,3	-0,130466	below	0,05457812	Not_Validated_STAR							0,96		
97	tested	TCGA-XF-A8HB-01A	BLCA	203	107	108,45	7,6	-0,141518	below	0,1121191	Not_Validated_STAR							0,89		
98	tested	TCGA-57-1993-01A	OV	266	101	127,11	10,2	-0,1902479	below	-0,0822064	Not_Validated_STAR							1		
99	tested	TCGA-E2-A15F-01A	BRCA	162	100	90,01	6,3	-0,2053725	below	0,00079815	Not_Validated_STAR							0,8		
100	notTested	TCGA-EY-A2OM-01A	UCEC	159	156	156,15	2,3		below		SBT_SF3B1_like		SUGP1_p.G566D_noLOH					0,81		
101	notTested	TCGA-CK-4951-01A	COAD	169	155	155,31	2,9		below		SBT_SF3B1_like	p.N1026S	SUGP1_p.A629T_noLOH					0,64		0,27
102	notTested	TCGA-AP-A051-01A	UCEC	141	136	136,50	2,4		below		SBT_SF3B1_like	p.R549C	new_causative_SF3B1_mutation	SF3B1_p.R549C				0,76		0,26
103	notTested	TCGA-AA-3506-01A	COAD	91	113	113,25	1		below		SBT_SF3B1_like						NA			
104	notTested	TCGA-AZ-4614-01A	COAD	106	103	102,64	2,3		below		SBT_SF3B1_like						NA			
105	tested	TCGA-FW-A3R5-06A	SKCM	182	99	96,17	7,1	-0,2159567	below	0,09477861					S15F			0,64		
106	notTested	TCGA-BS-A0UV-01A	UCEC	91	98	98,44	1,7		below			p.R957Q						0,84		0,29
107	notTested	TCGA-AZ-4684-01A	COAD	103	97	97,47	2,5		below								NA			
108	notTested	TCGA-AA-A01Z-01A	COAD	85	96	95,89	1,5		below								NA			
109	tested	TCGA-61-1918-01A	OV	239	95	113,71	9,4	-0,189269	below	-0,1111475								0,9		
110	tested	TCGA-CD-A4MJ-01A	STAD	465	94	211,67	18	-0,1266151	below	-0,0151923								0,8		
111	tested	TCGA-JY-A6F8-01A	ESCA	412	94	190,95	16	-0,1812829	below	0,19641401								1		
112	notTested	TCGA-AZ-4313-01A	COAD	82	92	92,22	1,6		below								NA			
113	notTested	TCGA-49-AARN-01A	LUAD	109	91	90,77	3,2		below								NA			
114	tested	TCGA-DI-A1BU-01A	UCEC	78	88	53,02	3,6	-0,2328509	below	-0,2182031		p.V155A				164_T/A		0,79		0,24
115	tested	TCGA-N5-A4RT-01A	UCS	173	85	84,43	7,3	-0,1777775	below	0,23284794		p.D102A						1		0,7
116	tested	TCGA-F1-6875-01A	STAD	281	85	120,85	11,4	-0,2396544	below	-0,0192073								0,92		
117	tested	TCGA-CC-A7IG-01A	LIHC	183	84	85,92	7,7	-0,2181652	below	-0,0539431								1		
118	tested	TCGA-BR-7707-01A	STAD	196	84	90,85	8,2	-0,225684	below	0,36493686								0,6		
119	tested	TCGA-SY-A9G5-01A	BLCA	90	81	52,18	4,3	-0,1804765	below	-0,1438992		p.E902K						0,9		0,34
120	tested	TCGA-ZF-AA4R-01A	BLCA	78	80	47,09	3,9	-0,196975	below	0,05317107		p.E902G						0,74		0,53
121	tested	TCGA-25-2398-01A	OV	210	80	92,65	8,9	-0,2086566	below	-0,0540269								0,7		
122	tested	TCGA-MX-A5UJ-01A	STAD	347	77	138,73	14,2	-0,14036	below	-0,1790709								0,45		
123	tested	TCGA-W3-AA1V-06B	SKCM	164	76	74,48	7,3	-0,1410155	below	0,0912748						133_R/C		0,88		
124	tested	TCGA-G2-AA3F-01A	BLCA	134	75	63,21	6,2	-0,2332991	below	-0,1670469		p.Q903P				17_D/H		0,9		0,11
125	tested	TCGA-GV-A3QI-01A	BLCA	191	74	82,11	8,4	-0,1614999	below	0,06211733								1		
126	tested	TCGA-RU-A8FL-01A	CRC	156	73	70,82	7,1	-0,19967	below	-0,0334286								0,87		
127	tested	TCGA-61-1907-01A	OV	238	71	96,85	10,3	-0,1497366	below	0,17376199								0,9		
128	tested	TCGA-ZF-A9RN-01A	BLCA	98	71	49,32	5	-0,2001144	below	0,130423						56_Q/E		0,9		
129	tested	TCGA-W3-AA1O-06A	SKCM	147	69	65,01	6,9	-0,2134397	below	0,22044529						156_R/H		0,87		
130	tested	TCGA-06-2569-01A	GBM	212	68	86,32	9,4	-0,2085122	below	-0,1699638								1		
131	tested	TCGA-FD-A62P-01A	BLCA	84	67	42,10	4,6	-0,1387198	below	0,05844396		p.E902K						0,5		0,25
132	tested	TCGA-86-A456-01A	LUAD	85	66	41,23	4,7	-0,2003039	below	-0,1752755						34_S/F		0,38		
133	tested	TCGA-GL-A59R-01A	KIRP	60	64	35,88	3,8	-0,2628734	below	-0,1018906		p.T916A						0,8		0,46
134	tested	TCGA-D9-A1X3-06A	SKCM	194	64	77,45	8,9	-0,1554647	below	0,1877367								1		
135	tested	TCGA-4Z-AA7Q-01A	BLCA	76	62	35,15	4,5	-0,1530437	below	-0,0297231		p.E902K						0,5		0,45
136	tested	TCGA-WC-A882-01A	UVM	64	60	29,63	4,1	-0,1597704	below	0,01471689					92-1_YGRPPDSHH/-		NA			
137	tested	TCGA-49-4488-01A	LUAD	56	60	26,58	3,8	-0,2226468	below	0,13472214								1		
138	tested	TCGA-WR-A838-01A	OV	330	60	121,65	14,2	-0,1699175	below	-0,177065		p.R318L					NA		NA	
139	tested	TCGA-86-8585-01A	LUAD	73	59	31,93	4,5	-0,2179144	below	-0,3185116						216_G/R		0,6		
140	tested	TCGA-AB-2932-03A	LAML	131	59	52,35	6,7	-0,1303377	below	-0,2457124							NA			
141	tested	TCGA-LN-A49V-01A	ESCA	403	59	165,81	17	-0,1986443	below	-0,077974								1		
142	tested	TCGA-AN-A0FY-01A	BRCA	216	57	80,48	10	-0,1789134	below	-0,0928463								0,9		
143	tested	TCGA-EE-A3AG-06A	SKCM	173	56	64,54	8,4	-0,2556564	below	0,15894001								0,95		
144	tested	TCGA-61-2109-01A	OV	260	56	93,62	11,7	-0,1915287	below	-0,1720251								0,9		
145	tested	TCGA-EK-A3GK-01A	CESC	193	55	70,47	9,2	-0,1772435	below	0,00838404					69_E/K			0,86		
146	tested	TCGA-V4-A9EM-01A	UVM	132	54	50,37	6,9	-0,1710484	below	0,1797304					92-99_YGRPPDSH/-		NA			
147	tested	TCGA-GV-A3QF-01A	BLCA	121	54	45,85	6,5	-0,1930464	below	-0,1407647		p.E902K						0,8		0,34
148	tested	TCGA-BR-8678-01A	STAD	348	54	123,99	15,1	-0,1551308	below	-0,0809133		p.I1241M						0,91	NA	
149	tested	TCGA-MA-AA41-01A	CESC	90	52	33,68	5,4	-0,200384	below	-0,041135		p.D470N						0,74		0,24
150	tested	TCGA-66-2773-01A	LUSC	113	51	41,65	6,3	-0,2327869	below	-0,1513322					179_S/C			0,51		
151	tested	TCGA-EW-A1PC-01B	BRCA	218	51	76,78	10,3	-0,1693329	below	0,00516202								0,96		
152	tested	TCGA-ZH-A8Y8-01A	CHOL	125	50	44,44	6,8	-0,180102	below	-0,014059								0,5		
153	tested	TCGA-BR-8289-01A	STAD	460	50	158,64	19,5	-0,1169534	below	-0,0487886								0,9		
154	tested	TCGA-55-6981-01A	LUAD	32	50	15,95	3,3	-0,2694396	below	0,06386003					47_R/L			0,5		
155	tested	TCGA-G2-A2EF-01A	BLCA	193	49	66,91	9,4	-0,2112968	below	-0,1170101								0,8		
156	tested	TCGA-W5-AA2Q-01A	CHOL	126	48	51,84	6,9	-0,2051761	below	0,0418268								1		
157	tested	TCGA-64-1680-01A	LUAD	59	47	20,36	4,4	-0,2026683	below	0,16863746						34_S/F		0,92		
158	tested	TCGA-ZF-A9RF-01A	BLCA	109	47	37,68	6,3	-0,1965487	below	-0,1984166						35_R/Q		0,7		
159	tested	TCGA-4Z-AA83-01A	BLCA	64	47	21,99	4,6	-0,2979768	below	-0,2791455								0,9		
160	tested	TCGA-ZF-AA51-01A	BLCA	61	47	19,52	4,5	-0,2139944	below	-0,0331639						124_E/K		0,74		
161	tested	TCGA-20-1684-01A	OV	391	47	137,67	17	-0,1823664	below	0,1404458								0,83		
162	tested	TCGA-AA-3660-01A	CRC	84	46	34,05	5,4	-0,1541699	below	-0,0982878		p.R16G						0,67		0,66
163	tested	TCGA-ER-A19F-06A	SKCM	115	45	37,38	6,6	-0,2292511	below	0,22609886		p.Y898H						0,95		0,23
164	tested	TCGA-55-1592-01A	LUAD	227	44	75,58	10,9	-0,1526103	below	-0,0131931								0,56		
165	tested	TCGA-FA-A86F-01A	DLBC	158	43	51,12	8,3	-0,1980339	below	-0,1086652								0,3		
166	tested	TCGA-18-3421-01A	LUSC	110	43	35,32	6,5	-0,1616291	below	0,07187932		p.K790N	SF3B1_RNAseq_notConfirmed					0,58		0,16
167	tested	TCGA-CL-4957-01A	READ	139	43	43,77	7,6	-0,1663475	below	0,18726619								0,72		
168	tested	TCGA-34-8455-01A	LUSC	94	43	28,92	5,9	-0,1750481	below	0,0254694		p.K111N						0,76		0,28
169	tested	TCGA-AN-A04D-01A	BRCA	197	43	64,80	9,8	-0,1169083	below	0,00294356								0,9		
170	tested	TCGA-N5-A4R8-01A	UCS	146	42	46,97	7,9	-0,2356602	below	0,27617459							NA			
171	tested	TCGA-A6-5665-01A	CRC	109	42	41,30	6,5	-0,1854426	below	0,07592151					48_D/G			0,85		
172	tested	TCGA-BF-A1PZ-01A	SKCM	175	42	55,66	9	-0,1936938	below	0,28436954		p.R614I	SF3B1_RNAseq_notConfirmed			111_G/V		0,95		0,1
173	tested	TCGA-BH-A0HY-01A	BRCA	114	42	35,39	6,7	-0,1543223	below	0,15081379		p.Y765C						0,92		0,24
174	tested	TCGA-22-4605-01A	LUSC	148	41	46,54	8	-0,0920836	below	-0,0022585		p.K700E	SF3B1_RNAseq_notConfirmed_5/290VAF					0,5		0,16
175	tested	TCGA-WC-A888-01A	UVM	74	41	21,43	5,2	-0,2091251	below	-0,0776216					173-179_SSSVSRS/S		NA			
176	tested	TCGA-AB-2868-03A	LAML	95	41	51,51	6	-0,1709102	below	-0,2123529		p.L833F					NA		NA	
177	tested	TCGA-EJ-5525-01A	PRAD	55	41	14,78	4,5	-0,1742894	below	-0,0754701					151-159_SRTRSRSRS/S			0,86		
178	tested	TCGA-68-7756-01A	LUSC	226	40	73,39	11	-0,0528098	below	-0,2176719								0,6		
179	tested	TCGA-56-A5DR-01A	LUSC	128	40	38,12	7,3	-0,1450024	below	0,01780372		p.Y765C						0,8		0,21
180	tested	TCGA-G3-A7M9-01A	LIHC	133	40	40,10	7,5	-0,1820142	below	0,14835268								0,9		
181	tested	TCGA-EE-A29V-06A	SKCM	191	40	60,53	9,7	-0,1834097	below	0,23687801								0,87		
182	tested	TCGA-VQ-A924-01A	STAD	183	39	57,38	9,4	-0,2192063	below	-0,053092					84_E/K			0,76		

183	tested	TCGA-ZB-A96P-01A	THYM	175	39	55,09	9,1	-0,2088127	below	-0,0820045					NA			
184	tested	TCGA-78-8655-01A	LUAD	67	37	15,75	5,1	-0,2414425	below	0,25661226				34_S/F		0,8		
185	tested	TCGA-A2-A0EN-01A	BRCA	136	37	36,77	8,4	-0,2662676	below	0,18045344		SUGP1_p.R642W_noLOH						
186	tested	TCGA-EE-A3AD-06A	SKCM	133	37	38,79	7,6	-0,1873444	below	0,17185718	p.P510Q	SF3B1_RNAseq_notConfirmed				0,87		0,12
187	tested	TCGA-GR-A4D4-01A	DLBC	138	37	39,27	7,8	-0,1985281	below	-0,0481605						0,7		
188	tested	TCGA-AB-2866-03A	LAML	93	37	24,63	6,1	-0,1965557	below	-0,1448791			95_P/H		NA			
189	tested	TCGA-ZH-A8Y5-01A	CHOL	135	36	38,96	7,7	-0,2183424	below	-0,0128484						1		
190	tested	TCGA-AJ-A3EK-01A	UCEC	103	36	28,28	6,5	-0,1950977	below	0,12265324	p.A368T					0,93		0,28
191	tested	TCGA-EI-6508-01A	READ	132	36	37,57	7,6	-0,2105846	below	0,21960047						0,95		
192	tested	TCGA-ZF-AA5P-01A	BLCA	84	36	20,77	5,8	-0,1789259	below	-0,1135051	p.L897R					0,2	NA	
193	tested	TCGA-2G-AAL5-01A	TGCT	150	35	42,76	8,3	-0,1950568	below	-0,3047525						1		
194	tested	TCGA-3H-AB3U-01A	MESO	131	35	36,08	7,6	-0,2207084	below	-0,0473922						1		
195	tested	TCGA-ZB-A96L-01A	THYM	165	35	47,43	8,9	-0,1394548	below	-0,2333011						1		
196	tested	TCGA-AJ-A3BD-01A	UCEC	91	35	22,10	6,1	-0,069524	below	0,17036423				24_I/T		0,9		
197	tested	TCGA-5P-A9K2-01A	KIRP	42	33	4,66	4,3	-0,3135094	below	-0,5018087	p.S461Y					0,78		0,44
198	tested	TCGA-JY-A6FA-01A	ESCA	335	33	104,84	15,4	-0,2246311	below	0,01583965						0,96		
199	tested	TCGA-CM-5861-01A	CRC	47	33	5,74	4,5	-0,2087613	below	0,29205127				97_E/D		0,95		
200	tested	TCGA-NA-A4QX-01A	UCS	150	33	42,44	8,4	-0,1215208	below	-0,096853						1		
201	tested	TCGA-55-A4DF-01A	LUAD	110	32	27,24	6,9	-0,1276186	below	-0,0777945						0,66		
202	tested	TCGA-4R-AA8I-01A	LIHC	54	32	7,67	4,8	-0,2409314	below	0,02561183	p.A433G					0,88		0,43
203	tested	TCGA-60-2719-01A	LUSC	141	32	37,83	8,1	-0,1683243	below	0,16968326	p.N185S					0,89		0,41
204	tested	TCGA-2G-AAGY-01A	TGCT	183	32	63,64	9,7	-0,1599624	below	-0,1827981						1		
205	tested	TCGA-DX-A7EN-01A	SARC	138	31	37,00	8	-0,1402313	below	0,06935221	p.A710T	SF3B1_RNAseq_notConfirmed				0,82		0,06
206	tested	TCGA-CA-6717-01A	CRC	90	31	19,49	6,2	-0,2324498	below	0,21002319	p.E722K					0,47		0,09
207	tested	TCGA-AB-2936-03A	LAML	119	31	28,98	7,3	-0,2042105	below	-0,3398697			95-12_PPDSHHSRR/R		NA			
208	tested	TCGA-VD-A8KJ-01A	UVM	140	31	37,11	8,1	-0,2041726	below	0,00897315						1		
209	tested	TCGA-GS-A9TQ-01A	DLBC	132	31	33,94	7,8	-0,1789594	below	-0,1474693						1		
210	tested	TCGA-X7-A8D7-01A	THYM	118	30	37,38	7,3	-0,2258423	below	-0,0299405					NA			
211	tested	TCGA-G9-A9S7-01A	PRAD	110	30	25,82	7	-0,2213365	below	-0,2432883				34_S/F		0,74		
212	tested	TCGA-L5-A8NN-01A	ESCA	300	30	90,53	14,2	-0,1582765	below	0,24363498						0,7		
213	tested	TCGA-DM-A0XF-01A	CRC	141	29	35,75	8,2	-0,2055081	below	0,04613576						0,68		
214	tested	TCGA-EX-A69L-01A	CESC	98	28	21,56	6,6	-0,2277484	below	-0,1167575				34_S/F		0,67		
215	tested	TCGA-AB-2825-03B	LAML	84	28	22,75	6,1	-0,1635691	below	0,08030498	p.T369N				NA			0,23
216	tested	TCGA-AB-2811-03B	LAML	150	28	37,42	8,6	-0,1183273	below	-0,2751904					NA			
217	tested	TCGA-AB-2891-03A	LAML	81	27	14,32	6	-0,1837879	below	-0,1707964	p.L833F	SF3B1_RNAseq_notConfirmed			NA			0
218	tested	TCGA-AB-2861-03B	LAML	86	27	23,14	6,2	-0,2084893	below	0,13915006				34_S/Y	NA			
219	tested	TCGA-64-1676-01A	LUAD	78	27	12,84	5,9	-0,0943654	below	0,21790659	p.A959V					0,5		0,29
220	tested	TCGA-N8-A56S-01A	UCS	125	26	29,06	7,7	-0,2061401	below	0,02619557				34_S/F	NA			
221	tested	TCGA-GC-A3BM-01A	BLCA	111	26	32,00	7,2	-0,1628099	below	-0,1099809	p.A176T					0,92		0,3
222	tested	TCGA-BH-A1EV-01A	BRCA	124	25	36,93	7,7	-0,2487626	below	0,0636845			136_S/C			0,9		
223	tested	TCGA-30-1718-01A	OV	306	25	90,52	14,6	-0,2180689	below	0,06087307						0,7		
224	tested	TCGA-36-1575-01A	OV	422	25	136,38	19	-0,1550957	below	-0,1753276						0,9		
225	tested	TCGA-EA-A3HS-01A	CESC	192	25	50,11	10,3	-0,1446223	below	-0,0796447						0,69		
226	tested	TCGA-KQ-A41N-01A	BLCA	99	24	19,09	6,8	-0,1217514	below	-0,3127765	p.R736S	SF3B1_RNAseq_notConfirmed				1	NA	
227	tested	TCGA-D9-A1JW-06A	SKCM	112	24	23,21	7,3	-0,210615	below	0,28813438	p.M620I	SF3B1_RNAseq_notConfirmed				0,57		0,08
228	tested	TCGA-GM-A5PX-01A	BRCA	75	24	10,52	5,9	-0,17791	below	-0,1549714				157_Q/R		0,67		
229	tested	TCGA-P6-A5OG-01A	ACC	101	23	19,21	6,9	-0,1634872	below	-0,2724627						1		
230	tested	TCGA-24-1463-01A	OV	394	23	140,67	18	-0,1708942	below	-0,0186019						0,83		
231	tested	TCGA-55-A493-01A	LUAD	77	23	10,01	6	-0,1898273	below	-0,1303788			218_A/T			0,2		
232	tested	TCGA-WE-A8K5-06A	SKCM	118	22	23,35	7,6	-0,227113	below	0,17043761	p.P351S					0,95		0,28
233	tested	TCGA-V4-A9EI-01A	UVM	139	22	30,73	8,4	-0,2196476	below	0,05037532						1		
234	tested	TCGA-06-0129-01A	GBM	202	21	51,60	10,8	-0,2130973	below	-0,1677489						1		
235	tested	TCGA-95-7039-01A	LUAD	46	21	-1,23	4,9	-0,2174739	below	-0,0170769			168_R/K			0,9		
236	tested	TCGA-24-1603-01A	OV	373	21	110,42	17,3	-0,1420611	below	-0,1518209						1		
237	tested	TCGA-G3-AAV1-01A	LIHC	69	21	4,94	5,8	-0,2261541	below	-0,0054019	p.A277S					1		0,39
238	tested	TCGA-G4-6586-01A	CRC	95	20	15,05	6,8	-0,2358868	below	0,14951916	p.R397C					0,83		0,31
239	tested	TCGA-EP-A3JL-01A	LIHC	87	20	12,28	6,5	-0,193636	below	0,10608537	p.V576A					0,8		0,26
240	tested	TCGA-UF-A7JS-01A	HNSC	31	19	-8,50	4,4	-0,3136141	below	0,40496361				34_S/Y		0,8		
241	tested	TCGA-XM-A8RE-01A	THYM	163	19	37,76	9,4	-0,2525186	below	-0,1862462					NA			
242	tested	TCGA-EO-A3AV-01A	UCEC	72	18	5,93	6	-0,2605146	below	0,17716251	p.L502F					0,2		0,26
243	tested	TCGA-69-7979-01A	LUAD	138	18	27,89	8,5	-0,2001318	below	-0,0139603	p.Q206R					1		0,39
244	tested	TCGA-W5-AA31-01A	CHOL	130	18	34,86	8,2	-0,2776592	below	-0,0688878						0,8		
245	tested	TCGA-GS-A9TT-01A	DLBC	159	18	34,94	9,3	-0,1843504	below	0,02972494						1		
246	tested	TCGA-ER-A19P-06A	SKCM	129	17	24,69	8,2	-0,1948288	below	-0,0194146	p.M620I	SF3B1_RNAseq_notConfirmed				0,19		0,14
247	tested	TCGA-DD-AADC-01A	LIHC	45	17	17,49	4	-0,3138739	below	0,04595163		SUGP1_p.G582Wfs*9_noLOH						
248	tested	TCGA-59-A5PD-01A	OV	348	17	98,72	16,5	-0,1264105	below	0,10337861					NA			
249	tested	TCGA-50-5941-01A	LUAD	52	17	-1,92	5,3	-0,2224104	below	0,11553254						0,3		
250	tested	TCGA-OR-A5LG-01A	ACC	102	17	14,92	7,2	-0,1946498	below	-0,2294881						1		
251	tested	TCGA-AJ-A3EL-01A	UCEC	94	16	12,45	6,9	-0,2374445	below	0,15301272	p.R1297C					0,91		0,49
252	tested	TCGA-E2-A1IO-01A	BRCA	115	16	17,91	7,7	-0,1508231	below	-0,1612016				156_R/H		0,58		
253	tested	TCGA-L6-A4EP-01A	THCA	78	16	6,84	6,3	-0,2295776	below	-0,3102423	p.M876L				NA			0,35
254	tested	TCGA-C5-A8YR-01A	CESC	70	16	3,81	6	-0,1362242	below	0,03307871			69_E/K			0,92		
255	tested	TCGA-DF-A2KU-01A	UCEC	82	16	16,24	6,4	-0,3139844	below	0,17966559		SUGP1_p.E262*_noLOH						
256	tested	TCGA-DQ-5631-01A	HNSC	193	15	45,02	10,7	-0,1963649	below	0,2696376						0,63		
257	tested	TCGA-AB-2943-03A	LAML	132	15	23,52	8,4	-0,1890727	below	-0,3312916						0,9		
258	tested	TCGA-85-6560-01A	LUSC	235	15	58,98	12,3	-0,1530486	below	0,20959562						0,62		
259	tested	TCGA-DD-AAEH-01A	LIHC	50	15	-4,43	5,3	-0,2075411	below	-0,0191336	p.V1138L					0,9		0,37
260	tested	TCGA-OR-A5J8-01A	ACC	97	14	10,58	7,1	-0,2182441	below	0,15592131	p.V184F				NA			0,58
261	tested	TCGA-OR-A5LC-01A	ACC	44	14	-7,94	5,1	-0,1976798	below	-0,2983696				24_I/T	NA			
262	tested	TCGA-L6-A4EQ-01A	THCA	86	14	6,55	6,7	-0,1738282	below	0,05688727				234_R/C	NA			
263	tested	TCGA-EO-A22U-01A	UCEC	91	13	7,88	6,9	-0,235592	below	-0,0106331	p.F1220V					0,84		0,23
264	tested	TCGA-B5-A5OE-01A	UCEC	149	13	29,28	9,1	-0,2294127	below	-0,0523602						0,88		
265	tested	TCGA-EE-A2MR-06A	SKCM	149	13	29,36	9,1	-0,2030043	below	-0,1131891						0,3		
266	tested	TCGA-50-8460-01A	LUAD	77	13	4,03	6,4	-0,2006718	below	0,02667024						0,49		
267	tested	TCGA-E7-A85H-01A	BLCA	111	12	14,07	7,7	-0,1747367	below	-0,0934855				1_G/A		0,91		
268	tested	TCGA-DK-A1A3-01A	BLCA	68	12	12,50		-0,2877286	below	0,04809845		SUGP1_p.M1?_noLOH						
269	tested	TCGA-CU-A5W6-01A	BLCA	97	12	9,53	7,2	-0,1362154	below	0,07498973	p.E902K					0,8		0,33
270	tested	TCGA-LK-A4O5-01A	MESO	144	11	24,98	9	-0,2057838	below	-0,1920492						1		
271	tested	TCGA-A4-A6HP-01A	KIRP	83	11	3,93	6,7	-0,2661419	below	-0,1941716	p.K121M					0,77		0,35
272	tested	TCGA-DA-A1HV-06A	SKCM	88	10	5,17	6,9	-0,1971288	below	0,19961103	p.H1256Y					0,83		0,5
273	tested	TCGA-D3-A8GM-06A	SKCM	117	10	14,80	8	-0,2079457	below	0,14099278	p.D564E					0,73		0,61
274	tested	TCGA-FD-A3B5-01A	BLCA	109	10	12,03	7,7	-0,2227915	below	0,16169954			25_R/L			1		

275	tested	TCGA-VD-AA8T-01A	UVM	135	10	20,67	8,7	-0,2098654	below	0,05792978						1	
276	tested	TCGA-IR-A3LA-01A	CESC	124	9	17,61	8,3	-0,2295351	below	-0,0759453				34_S/F		0,9	
277	tested	TCGA-AB-2899-03A	LAML	63	9	-3,13	6	-0,1028824	below	-0,3593232			95_P/H		NA		
278	tested	TCGA-DD-AAE3-01A	LIHC	39	9	-11,99	5,1	-0,302324	below	0,20273724	p.D321V					1	0,45
279	tested	TCGA-OR-A5JB-01A	ACC	102	9	9,55	7,5	-0,1785537	below	0,09156493	p.M1231T					1	0,35
280	tested	TCGA-WK-A8XX-01A	SARC	146	8	23,06	9,2	-0,2202131	below	0,06941935						0,93	
281	tested	TCGA-52-7812-01A	LUSC	85	7	2,83	6,9	-0,2220726	below	0,24771516	p.C965S					0,68	0,28
282	tested	TCGA-D3-A2JL-06A	SKCM	98	7	7,29	7,4	-0,2159904	below	-0,0913918	p.D894G					0,54	0,32
283	tested	TCGA-VQ-A8P2-01A	STAD	280	7	69,56	14,3	-0,1183395	below	0,24191772	p.V917M					0,88	0,39
284	tested	TCGA-V1-A9O5-01A	PRAD	103	7	17,54	7,6	-0,2413084	below	0,02101493						0,82	
285	tested	TCGA-FD-A6TC-01A	BLCA	116	7	12,38	8,1	-0,1862088	below	-0,1164037			215_E/K			0,74	
286	tested	TCGA-55-7283-01A	LUAD	79	7	0,68	6,7	-0,2249452	below	0,18076119				34_S/F		0,35	
287	tested	TCGA-MP-A4T4-01A	LUAD	84	6	1,94	6,9	-0,2250402	below	-0,2467111				34_S/F		0,2	
288	tested	TCGA-BL-A5ZZ-01A	BLCA	76	6	-0,43	6,6	-0,2489445	below	-0,0836616	p.G693D					0,4	0,34
289	tested	TCGA-3N-A9WC-06A	SKCM	76	6	-1,73	6,6	-0,2317121	below	0,22388242						0,57	
290	tested	TCGA-V4-A9F1-01A	UVM	134	6	18,85	8,8	-0,2318071	below	0,17680679						1	
291	tested	TCGA-FX-A8OO-01A	SARC	139	6	19,79	9	-0,1906527	below	-0,0341832						0,83	
292	tested	TCGA-Q1-A73R-01A	CESC	191	5	37,48	11	-0,184332	below	-0,0684214						0,35	
293	tested	TCGA-AB-2843-03A	LAML	51	5	-11,12	5,7	-0,2423487	below	0,03882724					NA		
294	tested	TCGA-06-5416-01A	GBM	146	5	21,42	9,3	-0,2236796	below	0,21535047	p.E862K			98_E/D		1	0,23
295	tested	TCGA-55-7907-01A	LUAD	35	5	-16,23	5,1	-0,2136923	below	-0,0767509						0,66	
296	tested	TCGA-AJ-A3NE-01A	UCEC	95	5	4,70	7,9	-0,2877398	below	0,25916971		SUGP1_p.R520W_noLOH					
297	tested	TCGA-BK-A6W3-01A	UCEC	130	5	16,89	8,7	-0,2464467	below	-0,1560135	p.C1035G					0,78	0,36
298	tested	TCGA-DM-A1HB-01A	CRC	106	5	7,20	7,8	-0,1644004	below	-0,0958413	p.P258L			198_R/Q		0,86	0,39
299	tested	TCGA-E7-A6ME-01A	BLCA	92	4	3,18	7,3	-0,1973719	below	-0,1335053				211_RG/R		0,92	
300	tested	TCGA-BT-A3PJ-01A	BLCA	113	4	9,22	8,1	-0,2375157	below	0,12280549			167_R/Q			0,74	
301	tested	TCGA-GR-A4D5-01A	DLBC	142	4	19,60	9,2	-0,2592529	below	-0,1402971						0,3	
302	tested	TCGA-30-1855-01A	OV	348	4	204,43	17	-0,1678341	below	0,10513001						0,7	
303	tested	TCGA-EE-A183-06A	SKCM	155	4	24,16	9,7	-0,2019944	below	0,17249841	p.R736S	SF3B1_RNAseq_notConfirmed	46_P/Q			0,35	0,11
304	tested	TCGA-51-4081-01A	LUSC	81	3	6,84	6,9	-0,2330851	below	-0,1047646						1	
305	tested	TCGA-QS-A5YQ-01A	UCEC	57	3	-9,67	6	-0,2391871	below	-0,3386641	p.R957Q					0,93	0,42
306	tested	TCGA-05-4422-01A	LUAD	210	3	42,60	11,8	-0,1612985	below	-0,2007706						0,71	
307	tested	TCGA-EO-A3AY-01A	UCEC	67	3	-6,45	6,4	-0,1831451	below	0,35572401	p.R315Q					0,77	0,36
308	tested	TCGA-AB-2810-03A	LAML	56	2	-10,76	6	-0,1825731	below	-0,2459156			95_P/H		NA		
309	tested	TCGA-A5-A2K5-01A	UCEC	82	2	-2,41	7	-0,1561818	below	0,34340298	p.K573T				NA		0,53
310	tested	TCGA-66-2777-01A	LUSC	82	2	-1,67	7	-0,2127	below	0,20356462				66_R/C		0,48	
311	tested	TCGA-AB-2948-03A	LAML	124	2	11,67	8,6	-0,1061274	below	-0,1850048			95_P/R_92_Y/YA		NA		
312	tested	TCGA-38-4630-01A	LUAD	219	1	44,98	12,2	-0,165426	below	-0,0210878						0,92	
313	tested	TCGA-DB-A75M-01A	LGG	84	1	-1,09	7,1	-0,2241321	below	0,15344517	p.D835V				NA		0,38
314	tested	TCGA-49-6744-01A	LUAD	68	1	0,30	6,5	-0,2263674	below	0,04504678						0,37	
315	tested	TCGA-SQ-A6I4-01A	PCPG	65	1	-1,09	6,4	-0,2564586	below	0,18006207						0,7	
316	tested	TCGA-AC-A5XS-01A	BRCA	80	0	-4,13	7	-0,2327928	below	0,0808644			52_K/N		NA		
317	tested	TCGA-C8-A134-01A	BRCA	209	-1	39,83	11,9	-0,1819426	below	0,10185186						0,7	
318	tested	TCGA-JW-A5VL-01A	CESC	100	-1	1,67	7,8	-0,2032499	below	0,17316089			14_G/E			0,8	
319	tested	TCGA-PE-A5DE-01A	BRCA	81	-2	-5,29	7,1	-0,2161656	below	-0,0435495			35_E/Q_1_M/I			0,56	
320	tested	TCGA-KN-8427-01A	KICH	117	-3	17,01	8,5	-0,1816656	below	0,09686408						1	
321	tested	TCGA-CM-5863-01A	CRC	72	-3	-9,26	6,8	-0,2241417	below	0,05275865	p.V727L					0,24	0,39
322	tested	TCGA-AB-2996-03A	LAML	72	-3	-8,43	6,8	-0,2031825	below	-0,2485276				34_S/F	NA		
323	tested	TCGA-23-1123-01A	OV	370	-3	93,15	18,1	-0,0960605	below	-0,0098777						1	
324	tested	TCGA-NH-A5IV-01A	CRC	108	-4	3,63	8,2	-0,2409907	below	-0,0396845	p.G922D					0,69	0,35
325	tested	TCGA-HU-A4H0-01A	STAD	398	-4	102,13	19,2	-0,1874487	below	0,12349197						0,46	
326	tested	TCGA-68-7757-01B	LUSC	81	-4	-7,27	7,2	-0,2583962	below	-0,2338468			162_K/R			0,38	
327	tested	TCGA-G4-6307-01A	CRC	144	-5	14,31	9,6	-0,1884106	below	-0,0868743						0,88	
328	tested	TCGA-S6-A8JY-01A	TGCT	159	-6	18,64	10,2	-0,2594703	below	0,01876947						1	
329	tested	TCGA-E6-A8L9-01A	UCEC	90	-6	-3,79	7,6	-0,2783495	below	-0,0947269			128_S/R			0,8	
330	tested	TCGA-AB-2865-03A	LAML	58	-6	-15,35	6,4	-0,1860461	below	-0,1427499			95_P/R		NA		
331	tested	TCGA-43-6143-01A	LUSC	240	-7	62,53	13,3	-0,2100605	below	0,06207621						0,89	
332	tested	TCGA-D3-A1Q3-06A	SKCM	179	-7	25,36	11	-0,1642702	below	0,22178085	p.R736S	SF3B1_RNAseq_notConfirmed				0,61	0,08
333	tested	TCGA-D7-6525-01A	STAD	369	-7	91,57	18,2	-0,1907343	below	0,04745844						0,76	
334	tested	TCGA-D3-A3CE-06A	SKCM	123	-7	5,46	8,9	-0,2458059	below	0,26735478				75_Q/H		0,8	
335	tested	TCGA-HM-A4S6-01A	CESC	91	-8	-5,14	7,7	-0,2245137	below	-0,1469123	p.D138N					0,5	0,3
336	tested	TCGA-E1-A7YU-01A	LGG	67	-8	-13,23	6,8	-0,2350267	below	0,07518932	p.V591E				NA		0,27
337	tested	TCGA-FR-A726-01A	SKCM	159	-8	17,13	10,3	-0,2429658	below	0,17520451	p.S229F					0,7	0,27
338	tested	TCGA-B5-A3FC-01A	UCEC	61	-9	-16,89	6,6	-0,2336607	below	-0,0537148	p.V977A		211_K/E			0,93	0,43
339	tested	TCGA-BR-8372-01A	STAD	359	-9	85,25	17,9	-0,1915973	below	0,06227945						0,69	
340	tested	TCGA-D6-A74Q-01A	HNSC	108	-9	-0,88	8,4	-0,1512522	below	-0,1638606			47_R/L			0,67	
341	tested	TCGA-AB-2941-03A	LAML	129	-9	7,18	9,2	-0,1814642	below	-0,3854959						0,5	
342	tested	TCGA-AB-3002-03A	LAML	68	-10	-13,98	6,9	-0,2070607	below	-0,3798788			95-12_PPDSHHSRR/R		NA		
343	tested	TCGA-US-A776-01A	PAAD	89	-10	-7,32	7,7	-0,2629395	below	-0,2514386	p.N763S					0,8	0,34
344	tested	TCGA-EE-A2GB-06A	SKCM	175	-11	20,76	11	-0,2172628	below	0,31102813						0,92	
345	tested	TCGA-EB-A3Y6-01A	SKCM	103	-12	-3,83	8,3	-0,1686848	below	0,19894683	p.P274L					0,8	0,3
346	tested	TCGA-85-A4CL-01A	LUSC	103	-12	-3,98	8,3	-0,2214486	below	-0,0638326			35_E/K			0,9	
347	tested	TCGA-DU-7008-01A	LGG	153	-12	13,42	10,2	-0,1549607	below	0,2235281	p.S541P					1	0,53
348	tested	TCGA-XE-A8H5-01A	TGCT	153	-12	12,89	10,2	-0,2106985	below	-0,039018						1	
349	tested	TCGA-KN-8424-01A	KICH	116	-12	11,17	8,8	-0,2140938	below	-0,0573474			69_E/G		NA		
350	tested	TCGA-EB-A4IS-01A	SKCM	108	-12	-2,10	8,5	-0,2327609	below	0,13186392			94_R/C			0,51	
351	tested	TCGA-AB-2847-03A	LAML	81	-12	-11,63	7,5	-0,2079985	below	-0,2271872					NA		
352	tested	TCGA-46-6025-01A	LUSC	213	-12	34,19	12,5	-0,0836702	below	0,04763455						0,7	
353	tested	TCGA-SG-A6Z4-01A	SARC	157	-13	14,43	10,4	-0,1144316	below	-0,1659006						0,92	
354	tested	TCGA-DU-7302-01A	LGG	67	-13	-16,61	7	-0,2291905	below	0,25092841			172-173_KS/-		NA		
355	tested	TCGA-ZJ-AAXU-01A	CESC	148	-14	9,30	10,1	-0,1782678	below	-0,0199908			215_E/K_167_R/Q			0,62	
356	tested	TCGA-Q9-A6FU-01A	ESCA	383	-14	113,52	19	-0,1461814	below	0,22833476						0,8	
357	tested	TCGA-78-7145-01A	LUAD	49	-15	-24,06	6,4	-0,2314839	below	0,22941333				34_S/F		0,9	
358	tested	TCGA-AB-2990-03B	LAML	107	-15	-4,16	8,6	-0,1913592	below	-0,3190697			95_P/L		NA		
359	tested	TCGA-24-1424-01A	OV	379	-15	89,46	18,9	-0,16908	below	0,02144173						0,9	
360	tested	TCGA-DM-A1DA-01A	CRC	146	-16	8,43	10,1	-0,1558561	below	-0,1082242						0,65	
361	tested	TCGA-EE-A181-06A	SKCM	109	-16	-4,45	8,7	-0,2468173	below	0,03248263	p.P812S					0,6	0,29
362	tested	TCGA-22-5480-01A	LUSC	172	-16	16,01	11,1	-0,2178265	below	-0,0732546	p.A1047S					0,76	0,28
363	tested	TCGA-P6-A5OF-01A	ACC	111	-17	-4,30	8,8	-0,2780924	below	-0,2712359					NA		
364	tested	TCGA-CX-7219-01A	HNSC	113	-17	-3,89	8,9	-0,2273821	below	0,06531411				38_N/H		0,49	
365	tested	TCGA-AX-A2HH-01A	UCEC	102	-18	-8,56	8,5	-0,2125188	below	0,16661916				34_S/F		0,84	
366	tested	TCGA-CV-6948-01A	HNSC	189	-18	22,32	11,8	-0,143471	below	0,06773934						0,85	

367	tested	TCGA-D9-A4Z2-01A	SKCM	120	-18	-1,53	9,2	-0,25586	below	0,00879213			149_S/P			0,89		
368	tested	TCGA-AC-A23H-01A	BRCA	141	-18	16,80	10	-0,1756875	below	0,19187951	p.L464F					0,74		0,33
369	tested	TCGA-CZ-5465-01A	KIRC	201	-19	39,67	12,3	-0,068502	below	-0,0294471						0,58		
370	tested	TCGA-L5-A4OF-01A	ESCA	404	-20	102,24	20	-0,2149039	below	-0,0858008						0,9		
371	tested	TCGA-SL-A6JA-01A	UCEC	87	-20	-14,13	8	-0,3020243	below	0,05987173				166_G/V		0,93		
372	tested	TCGA-EJ-5511-01A	PRAD	167	-21	11,92	11,1	-0,2558182	below	-0,1238357						0,8		
373	tested	TCGA-FS-A4F5-06A	SKCM	122	-22	-4,32	9,4	-0,2172536	below	0,07576509	p.R1297C					0,95		0,41
374	tested	TCGA-G2-A2EO-01A	BLCA	76	-23	-19,81	7,7	-0,1770007	below	0,00141189			129_R/L			0,6		
375	tested	TCGA-KN-8430-01A	KICH	89	-23	-6,14	8,2	-0,1919188	below	0,03757324						1		
376	tested	TCGA-UZ-A9PS-01A	KIRP	81	-23	-9,30	7,9	-0,2476232	below	0,10360435	p.T935K					0,85		0,21
377	tested	TCGA-HU-8608-01A	STAD	260	-24	41,66	14,7	-0,2277665	below	0,14876601				182_R/Q		0,46		
378	tested	TCGA-AG-3902-01A	READ	149	-24	4,32	10,5	-0,1711913	below	-0,1577479						1		
379	tested	TCGA-GR-7351-01A	DLBC	59	-24	-26,53	7,1	-0,2207709	below	0,32913876	p.T434A				NA			0,28
380	tested	TCGA-IB-7651-01A	PAAD	129	-24	-24,36	11,8	-0,2850775	below	0,18552407		SUGP1_p.E533K_noLOH						
381	tested	TCGA-DX-A6YV-01A	SARC	56	-24	-28,09	7	-0,183729	below	0,03885728	p.Q1248K	SF3B1_RNAseq_notConfirmed				0,92		0,2
382	tested	TCGA-55-7903-01A	LUAD	92	-25	-16,83	8,4	-0,1992203	below	0,0307133				34_S/F		0,72		
383	tested	TCGA-DA-A1I1-06A	SKCM	95	-27	-16,98	8,6	-0,2015102	below	0,21397425			51_T/I			0,67		
384	tested	TCGA-BH-A1F0-01A	BRCA	216	-28	40,15	13,2	-0,0881243	below	0,07984429						0,92		
385	tested	TCGA-E8-A433-01A	THCA	76	-28	-23,99	7,9	-0,264513	below	0,03268465	p.T7I				NA			0,31
386	tested	TCGA-DX-A3LU-01A	SARC	60	-28	-30,00	7,3	-0,1461448	below	0,03189258	p.F746V	SF3B1_RNAseq_notConfirmed_lowCoverage				0,73		0,09
387	tested	TCGA-D8-A1JA-01A	BRCA	122	-29	-29,01	11,7	-0,2780782	below	0,08999499		SUGP1_p.M1?_noLOH						
388	tested	TCGA-KN-8423-01A	KICH	125	-29	4,03	9,8	-0,1964238	below	0,13132975						1		
389	tested	TCGA-DU-6407-01A	LGG	74	-30	-16,30	7,9	-0,2819617	below	0,34674656	p.L464F					1		0,16
390	tested	TCGA-GN-A26C-01A	SKCM	105	-31	-14,82	9,1	-0,2427962	below	0,1228646	p.M620I	SF3B1_RNAseq_notConfirmed				0,5		0,1
391	tested	TCGA-DM-A1DB-01A	CRC	144	-31	-2,19	10,6	-0,1992533	below	-0,0623223						0,95		
392	tested	TCGA-AL-3472-01A	KIRP	99	-31	-18,22	8,9	-0,2233141	below	-0,4748948	p.V1169I					0,88		0,4
393	tested	TCGA-D6-6516-01A	HNSC	88	-32	-21,26	8,5	-0,1504395	below	0,33574045			167_R/L			0,69		
394	tested	TCGA-DW-7834-01A	KIRP	77	-32	-25,32	8,1	-0,1781027	below	-0,068237	p.T267N					0,92		0,35
395	tested	TCGA-KN-8426-01A	KICH	132	-33	4,57	10,2	-0,2484668	below	-0,1023528					NA			
396	tested	TCGA-33-4532-01A	LUSC	145	-33	-3,28	10,7	-0,2071049	below	0,00764218	p.A86T					1		0,34
397	tested	TCGA-HZ-7924-01A	PAAD	155	-36	-1,67	11,2	-0,2370519	below	-0,1666947						0,93		
398	tested	TCGA-B0-4816-01A	KIRC	139	-36	-8,13	10,6	-0,2091095	below	-0,203784	p.D1175Y					0,5	NA	
399	tested	TCGA-99-7458-01A	LUAD	99	-37	-21,22	9,1	-0,2209761	below	0,28538322				34_S/F		0,4		
400	tested	TCGA-V5-A7RB-01A	ESCA	281	-37	40,24	16	-0,1784016	below	0,11550442	p.T1113A					0,77		0,34
401	tested	TCGA-IB-8127-01A	PAAD	170	-37	3,51	11,8	-0,1813255	below	-0,1817461						0,9		
402	tested	TCGA-CC-A1HT-01A	LIHC	154	-37	-2,35	11,2	-0,2148631	below	-0,0108499						0,5		
403	tested	TCGA-FJ-A871-01A	BLCA	100	-38	-22,77	9,2	-0,2542491	below	-0,003321	p.N185S					0,78		0,38
404	tested	TCGA-CV-6962-01A	HNSC	81	-39	-18,99	8,5	-0,2109582	below	0,2599785			129_R/G			0,85		
405	tested	TCGA-AB-2959-03A	LAML	73	-42	-33,75	8,3	-0,3063155	below	-0,3833096			95_P/L		NA			
406	tested	TCGA-46-6026-01A	LUSC	205	-42	12,09	13,3	-0,1628662	below	0,07017314						0,9		
407	tested	TCGA-AH-6549-01A	READ	131	-44	-15,23	10,6	-0,2423766	below	-0,013404					NA			
408	tested	TCGA-AT-A5NU-01A	KIRP	181	-44	1,63	12,5	-0,2285743	below	-0,1771962						0,76		
409	tested	TCGA-34-5239-01A	LUSC	210	-45	11,35	13,6	-0,1650837	below	-0,1148972						0,8		
410	tested	TCGA-HT-8104-01A	LGG	96	-45	-27,52	9,3	-0,2632802	below	0,25070319	p.I665F					1		0,16
411	tested	TCGA-CN-6023-01A	HNSC	198	-46	6,78	13,2	-0,2009145	below	0,15770275						0,41		
412	tested	TCGA-D7-5578-01A	STAD	185	-48	0,47	12,8	-0,2489322	below	-0,0245898						0,7		
413	tested	TCGA-AB-2933-03A	LAML	58	-49	-43,10	8	-0,2341213	below	-0,1292334			95_P/L		NA			
414	tested	TCGA-44-7671-01A	LUAD	113	-49	-24,70	10,1	-0,2063584	below	0,18961614						0,74		
415	tested	TCGA-AR-A251-01A	BRCA	216	-49	10,75	14	-0,1948939	below	0,00793427						0,9		
416	tested	TCGA-32-2634-01A	GBM	214	-51	9,21	14	-0,2072495	below	0,01046174						1		
417	tested	TCGA-SW-A7EA-01A	STAD	174	-51	-5,40	12,5	-0,1942792	below	0,28821628				188_R/H		0,64		
418	tested	TCGA-E2-A1B0-01A	BRCA	213	-52	8,33	14	-0,2085152	below	0,09332853						0,66		
419	tested	TCGA-AB-2912-03A	LAML	67	-53	-43,63	8,5	-0,2740893	below	-0,2724631	Splice_Site	SF3B1_RNAseq_notConfirmed		34_S/F		1		0,12
420	tested	TCGA-CV-6950-01A	HNSC	132	-54	-22,30	11	-0,2346207	below	0,04462196	p.Y101C					0,62		0,62
421	tested	TCGA-E9-A1R5-01A	BRCA	100	-54	-32,19	9,8	-0,2387343	below	0,07034102	p.T1096K					0,79		0,31
422	tested	TCGA-A8-A09I-01A	BRCA	210	-55	4,45	14	-0,1994833	below	0,18122775						0,86		
423	tested	TCGA-AB-2826-03A	LAML	48	-56	-51,36	7,9	-0,2226942	below	0,07040595			95_P/H		NA			
424	tested	TCGA-BQ-7061-01A	KIRP	127	-56	-11,73	10,9	-0,2346852	below	0,24867317						0,75		
425	tested	TCGA-A6-6648-01A	CRC	145	-57	-18,63	11,6	-0,197917	below	0,10975757						0,51		
426	tested	TCGA-A4-A5Y1-01A	KIRP	174	-59	-11,11	12,8	-0,1887982	below	-0,0427668						0,89		
427	tested	TCGA-BR-A4PE-01A	STAD	337	-60	217,64	19	-0,190081	below	0,06761575						0,9		
428	tested	TCGA-69-8255-01A	LUAD	207	-61	0,03	14,1	-0,1809464	below	-0,0665888						0,58		
429	tested	TCGA-EE-A2GE-06A	SKCM	93	-61	-39,69	9,8	-0,2437201	below	0,01231593				119_R/S		0,3		
430	tested	TCGA-US-A77E-01A	PAAD	78	-63	-45,71	9,3	-0,2584339	below	-0,2596416				34_S/F		0,42		
431	tested	TCGA-23-1032-01A	OV	387	-63	85,24	21	-0,0886506	below	0,16818235						0,86		
432	tested	TCGA-DD-AADV-01A	LIHC	87	-64	-43,61	9,7	-0,231265	below	0,09793256	p.A711V					1		0,45
433	tested	TCGA-12-3653-01A	GBM	92	-65	-42,65	9,9	-0,2537852	below	0,22300621	p.W388G				NA			0,22
434	tested	TCGA-AB-2917-03A	LAML	68	-65	-49,92	9	-0,1789072	below	-0,3536721			95_P/H		NA			
435	tested	TCGA-DU-8168-01A	LGG	146	-66	-24,98	12	-0,2053429	below	0,18504468						1		
436	tested	TCGA-06-0745-01A	GBM	230	-67	3,90	15,2	-0,1804442	below	0,01447104						1		
437	tested	TCGA-AB-2975-03A	LAML	54	-68	-57,93	8,6	-0,1862908	below	-0,2337762			95_P/L		NA			
438	tested	TCGA-DU-6392-01A	LGG	120	-68	-35,72	11,1	-0,2132149	below	0,33335699	p.F814L			28_R/H		0,7		0,11
439	tested	TCGA-H2-A2K9-01A	THCA	201	-69	9,00	14,2	-0,177704	below	-0,1102509						0,3		
440	tested	TCGA-EJ-7791-01A	PRAD	161	-70	-21,13	12,7	-0,2296666	below	-0,0638996						0,94		
441	tested	TCGA-HZ-8519-01A	PAAD	152	-73	-28,15	12,5	-0,214246	below	0,07381248						0,76		
442	tested	TCGA-CG-4460-01A	STAD	99	-74	-45,87	10,5	-0,2664387	below	-0,039425	p.V1169I					0,79		0,3
443	tested	TCGA-49-4505-01A	LUAD	128	-74	-35,08	11,6	-0,2060692	below	0,18617176						0,54		
444	tested	TCGA-EL-A3ZQ-01A	THCA	46	-76	-55,60	8,6	-0,3055504	below	0,02201384					NA			
445	tested	TCGA-E2-A1B1-01A	BRCA	212	-77	-8,92	14,9	-0,1562833	below	-0,1052573						0,35		
446	tested	TCGA-09-2044-01B	OV	248	-78	3,34	16,3	-0,2200952	below	0,17495907	p.Q737K	SF3B1_RNAseq_notConfirmed		64_G/C		0,89		0,11
447	tested	TCGA-34-5929-01A	LUSC	215	-79	-10,26	15,1	-0,1798551	below	0,11459276						0,92		
448	tested	TCGA-KP-A3W1-01A	UCEC	66	-80	-62,16	9,5	-0,2718694	below	-0,249425				34_S/F		0,9		
449	tested	TCGA-A6-6780-01A	CRC	91	-82	-41,27	10,5	-0,2077031	below	0,05081825	p.L575P	SF3B1_RNAseq_notConfirmed				0,68		0,05
450	tested	TCGA-HZ-8637-01A	PAAD	122	-85	-45,00	11,8	-0,2118004	below	-0,0209967				34_S/F		0,1		
451	tested	TCGA-BR-7851-01A	STAD	247	-87	16,26	16,6	-0,2172592	below	-0,0374667	p.Q518R					0,72		0,22
452	tested	TCGA-XF-AAN3-01A	BLCA	186	-87	-24,63	14,3	-0,2102899	below	-0,0557484	p.E860K					0,37		0,35
453	tested	TCGA-C5-A1BJ-01A	CESC	198	-88	-21,69	14,8	-0,2351762	below	-0,2011958						0,41		
454	tested	TCGA-CG-5721-01A	STAD	206	-91	-2,14	15,2	-0,1956666	below	0,09152065	p.L544P					0,93		0,24
455	tested	TCGA-L5-A43C-01A	ESCA	210	-103	-7,82	15,8	-0,2142036	below	-0,1329529	p.Q669K	SF3B1_RNAseq_notConfirmed	95_P/L			0,4		0,06
456	tested	TCGA-IB-7654-01A	PAAD	99	-103	-64,58	11,6	-0,2515089	below	0,06728019				34_S/F		0,52		
457	tested	TCGA-CH-5767-01A	PRAD	101	-114	-57,10	12,1	-0,1717483	below	-0,0910692						0,92		
458	tested	TCGA-BA-4078-01A	HNSC	182	-117	-45,43	15,3	-0,1363275	below	0,01624479						0,88		

459	tested	TCGA-VQ-A91S-01A	STAD	276	-121	-16,40	19	-0,1301956	below	0,00530163	p.P327R	NA	0,37
460	tested	TCGA-QR-A7IP-01A	PCPG	117	-122	-70,49	13	-0,2124588	below	0,02630121		1	
461	tested	TCGA-A6-3809-01A	CRC	51	-132	-87,73	10,9	-0,2672487	below	0,14427147	p.S49I	0,87	0,27
462	tested	TCGA-QT-A5XO-01A	PCPG	122	-143	-82,95	14	-0,1701516	below	0,14730293		1	
463	tested	TCGA-D8-A27V-01A	BRCA	140	-146	-80,10	14,8	-0,1874806	below	-0,0534536	p.S455X	0,8	NA
464	tested	TCGA-DD-AACK-01A	LIHC	131	-158	-90,63	14,9	-0,225193	below	0,27260115		0,92	
465	tested	TCGA-FE-A233-01A	THCA	196	-169	-75,12	17,8	-0,2135523	below	0,14908206		NA	
466	tested	TCGA-BL-A0C8-01A	BLCA	101	-233	-129,74	16,6	-0,2576115	below	0,04680603	p.E902K	0,9	0,71
467	tested	TCGA-24-1428-01A	OV	386	-389	-154,19	33,3	-0,1814489	below	-0,0781045		0,73	
